# Study on the anti-inflammatory effects and mechanisms of gentisic acid based on the LPS-induced RAW264.7 cell inflammation model and the oxazolone-induced zebrafish inflammation model

**DOI:** 10.3389/fphar.2026.1837686

**Published:** 2026-06-26

**Authors:** Yang Han, Weiwei Zhou, Zufeng Zhang, Yuxin Zhang, Ling Liu, Jinhua Zhao, Wei Lyu, Xiumei Li

**Affiliations:** 1 Department of Infectious Diseases, Pekin Union Medical College Hospital, Beijing, China; 2 Institute of Feed Research of Chinese Academy of Agricultural Sciences, Beijing, China

**Keywords:** anti-inflammatory effect, experimental validation, gentisic acid, IL-17/NF-κB signaling pathway, intestinal microbiota, network pharmacology, zebrafish

## Abstract

**Background:**

Gentisic acid (GA), a natural polyphenolic compound, possesses significant anti-inflammatory activity, but its molecular mechanism, particularly its association with the intestinal microbiota and specific signaling pathways, remains unclear.

**Purpose:**

In this study, we aimed to clarify anti-inflammatory targets, pathways and mechanism of GA using network pharmacology, *in vitro* and *in vivo* experiments, and microbiome analysis.

**Materials and methods:**

Network pharmacology was used to predict the anti-inflammatory targets and pathways of GA. Lipopolysaccharide (LPS)-induced RAW 264.7 cell models and oxazolone (OXA)-induced zebrafish models were established to evaluate the anti-inflammatory effects of GA. Quantitative real-time polymerase chain reaction (qRT-PCR), Western blotting, and 16S rDNA sequencing were used to detect changes in key genes, proteins, and intestinal microbiota, respectively.

**Results and discussion:**

GA significantly attenuated LPS- and OXA-induced inflammation, reducing nitric oxide (NO) levels and inflammatory cytokines (IL-1β, TNF-α, and IL-6) in RAW 264.7 cells, along with IL-6 and IL-1β levels in zebrafish intestinal tissues. It was associated with the regulation of the IL-17 signaling pathway, reducing the abundance of *Acinetobacter*, *Allorhizobium–Neorhizobium–Pararhizobium–Rhizobium*, and *Shewanella*, which were positively correlated with key genes (IKKα, IKKβ, TRAF6, etc.) in the IL-17 pathway.

**Conclusion:**

GA may exert anti-inflammatory effects potentially via correlatively regulating the composition of pathogenic intestinal bacteria to maintain microecological homeostasis and modulating the IL-17/NF-κB pathway. These correlative findings provide a valuable reference for the research and development of polyphenolic compounds.

## 1 Introduction

External stimuli or tissue injury can activate immune cells to trigger the inflammatory response, a fundamental immune process sustaining tissue homeostasis and defending against pathogenic infection (Mia and Singh, 2022; Pérez-Pérez et al., 2020). Dysregulated inflammation, either inadequate or excessive, facilitates pathogen invasion and the onset of multiple disorders, such as arthritis, atherosclerosis, and cancer (Zanoli, 2019; Sharma et al., 2020). Non-steroidal anti-inflammatory drugs (NSAIDs) and glucocorticoids are the most commonly used anti-inflammatory drugs that rapidly ameliorate inflammation and block inflammatory cascades (Panchal and Prince, 2023). Nevertheless, their long-term overuse causes adverse effects, including gastrointestinal injury, hepatorenal toxicity, immune dysfunction, and drug resistance (Bindu et al., 2020; Chang et al., 2021; Hijos-Mallada et al., 2022). Moreover, most synthetic anti-inflammatory drugs act via a single target and fail to improve chronic inflammation-related intestinal microecological imbalance and mucosal damage, limiting their long-term clinical application and safety (Vega-Morales et al., 2021).

Against this background, natural bioactive substances with high safety, low toxicity, and multi-target regulatory effects have gradually become a research hotspot in the field of anti-inflammation. Among them, polyphenolic compounds possess multiple biological properties, including anti-inflammatory, antioxidant, and intestinal protective activities (Chen et al., 2020). Polyphenols are secondary metabolites of plants and can be divided into flavonoids, tannins, terpenoids, and lignans (Yu et al., 2022). Polyphenolic compounds contain at least one phenolic ring, and the number and position of their phenolic hydroxyl groups are crucial to their efficacy. More than 8,000 different structures of polyphenols are known, and hundreds of them have been isolated from natural plants, such as vegetables, fruits, nuts, tea, and herbs (Bolat et al., 2024). Gentisic acid (2,5-dihydroxybenzoic acid, GA) is a natural phenolic acid compound that is widely distributed in plants of the *Gentianaceae*, *Lamiaceae*, *Asteraceae*, and other families and can also be obtained via chemical synthesis. It is not only an important metabolite of the nonsteroidal anti-inflammatory drug aspirin but also a secondary metabolite found in plants such as gentiana, lemon, and sesame, as well as in fruits such as kiwi, apple, and pear (Abedi et al., 2020). Extensive research has demonstrated that GA possesses anti-inflammatory, antimutagenic, hepatoprotective, neuroprotective, antibacterial, and antioxidant activities (Dong et al., 2022; Han et al., 2021; Kang et al., 2021; Pujari and Bandawane, 2021). Nevertheless, the anti-inflammatory activity and molecular regulatory mechanisms of GA are still insufficiently characterized, in particular its functional correlation with gut microbiota and relevant signaling networks.

Lipopolysaccharide (LPS), a key component of Gram-negative bacterial cell walls and a typical pro-inflammatory mediator abundant in intestinal microbiota (Candelli et al., 2021), specifically binds to TLR4 on immune cells, activating canonical inflammatory pathways (e.g., NF-κB and MAPK). This promotes pro-inflammatory cytokine (TNF-α and IL-6) secretion, triggering inflammatory cascade amplification; sustained LPS exposure drives chronic inflammation progression (Zhou et al., 2024). The LPS-stimulated RAW 264.7 macrophage model, with high stability and clear mechanism, is a classic *in vitro* system for anti-inflammatory bioactivity screening (Facchin et al., 2022). Oxazolone (OXA), the first chemically induced enterocolitis model in zebrafish, specifically mimics Th2-type immune response-related pathological features of human ulcerative colitis (UC) (Brugman et al., 2009). As a well-recognized *in vivo* model, it recapitulates intestinal mucosal injury and immune infiltration, and its advantages (high zebrafish–human genetic homology, intuitive observation, short cycle, and controllable cost) enable the rapid evaluation of anti-inflammatory and intestinal protective effects (Schneider et al., 2024). Our team has successfully established and optimized this model, providing reliable technical support for *in vivo* pharmacological verification (Yang et al., 2023a; Zhou et al., 2026). These two complementary models clarify anti-inflammatory target pathways at the cellular and molecular levels and verify *in vivo* efficacy. Network pharmacology, an emerging interdisciplinary technology integrating systems biology, bioinformatics, and pharmacology, screens core targets and key signaling pathways via database mining and bioinformatics analysis based on the “drug–target–disease” interaction network, revealing drugs’ multi-target and multi-pathway synergistic regulatory mechanisms (Zhang et al., 2023). Compared with traditional pharmacology, it is efficient, rapid, and comprehensive, predicting potential targets of natural bioactive compounds and their associations with inflammation-related targets to support subsequent experiments, and is particularly suitable for studying multi-target natural products, such as polyphenols (Merecz-Sadowska et al., 2025).

Therefore, we first adopted a network pharmacology approach to predict the anti-inflammatory targets and related signaling pathways of GA. Subsequently, it systematically evaluated its anti-inflammatory activity by establishing RAW 264.7 macrophage inflammation models and zebrafish intestinal inflammation models and further explored correlative relationships between intestinal flora variation and inflammatory pathway expression by combining technical approaches including quantitative real-time polymerase chain reaction (qRT-PCR), Western blotting, intestinal microbiota sequencing analysis, and correlation analysis. This study not only provides a solid theoretical basis for an in-depth elucidation of the anti-inflammatory mechanisms of GA but also offers a valuable experimental reference for the development and utilization of natural polyphenolic compounds.

## 2 Materials and methods

### 2.1 Materials

High-glucose Dulbecco’s modified Eagle’s medium (DMEM), penicillin, streptomycin, and fetal bovine serum (FBS) were purchased from Gibco (United States). DMSO and LPS from *Escherichia coli* O127:B8 were purchased from Sigma (United States). GA was purchased from Macklin (Shanghai, China). The CCK8 kit, LDH kit, NO assay kit, BCA protein assay kit, Cell lysis buffer for Western and IP, and protease inhibitor cocktail for general use were purchased from Beyotime Biotechnology (Shanghai, China). TransScript® First-Strand cDNA Synthesis SuperMix was purchased from TransGen Biotech (Beijing, China). FastFire qPCR PreMix (SYBR Green) was purchased from TianGen (Beijing, China). The TGX™ FastCast™ acrylamide kit was purchased from Bio-Rad (Berkeley, California, United States).

Antibodies against GAPDH, TNFR1, TRAF2, IL-17A, TRAF6, IKKα, IKKβ, and p-p65 were purchased from CST (United States). Antibodies against p65, LCK, and zap70 were purchased from Proteintech (Wuhan, China). The IRDye 680RD goat anti-rabbit secondary antibody and the IRDye 800RD goat anti-mouse secondary antibody were purchased from LI-COR (Lincoln, NE, United States).

### 2.2 Network pharmacology analysis

The HERB database (http://herb.ac.cn/), STITCH database (http://stitch.embl.de/), TCMSP database (https://www.tcmsp-e.com/), and BATMAN-TCM database (http://bionet.ncpsb.org.cn/batman-tcm/) were utilized to predict the targets of GA action (Xia et al., 2022). The GeneCards databases (https://www.genecards.org/), OMIM databases (https://www.omim.org/), and NCBI databases (https://www.ncbi.nlm.nih.gov/) were used to screen the targets associated with inflammation (Stelzer et al., 2016).

The intersecting targets were imported into the STRING database (https://string-db.org) to establish a protein–protein interaction (PPI) network (Szklarczyk et al., 2017), and then, the data were analyzed and visualized using Cytoscape 3.8.0 software. All core targets were imported into the DAVID database (https://david.ncifcrf.gov/) for enrichment analysis of GO and KEGG (Yu et al., 2012). Further identification of compounds corresponding to key targets was carried out, and validation experiments were conducted on the top 10 key targets.

### 2.3 Cell viability

#### 2.3.1 RAW264.7 cell culture

RAW264.7 cells were cultured in DMEM containing 10% FBS, 100 U/mL penicillin, and 100 μg/mL streptomycin. The cell line was incubated at 37 °C in a humidified atmosphere with 5% CO_2_, and the culture medium was changed every 2 days. Subculturing was performed when the cells reached 80% confluence.

#### 2.3.2 RAW264.7 cell viability assays

To evaluate the effects of GA on the viability and cytotoxicity of RAW 264.7 cells, CCK-8 and LDH release assays were performed under unified culture conditions. In brief, RAW 264.7 cells were seeded onto 96-well plates at 1 × 10^5^ cells/well and cultured for 24 h for attachment and stabilization. The gradient concentrations of GA (6.25, 12.5, 25, 50, and 100 μg/mL) were identical in both assays.

For the CCK-8 assay, three groups were set: blank control (medium only without cells), normal control (cells without GA treatment), and GA-treated groups (cells incubated with the above gradient concentrations of GA). After 24 h of cell stabilization, cells in GA-treated groups were exposed to serially diluted GA for another 24 h. Subsequently, 10 μL of the CCK-8 reagent was added to each well under light-protected conditions, followed by incubation at 37 °C for 1 h. The absorbance at 450 nm was measured, and cell viability was calculated as follows: survival rate (%) = (OD_sample_ − OD_blank_) / (OD_control_ − OD_blank_) × 100% (Li et al., 2022).

For the LDH release assay, five groups were set: background blank control (medium only), sample control (cells and medium), maximum enzyme activity control (cells and medium), GA treatment (cells, medium, and GA), and compound blank control (medium and GA). After 24 h of stabilization, the supernatant was removed, and each well was rinsed once with PBS. An aliquot of 200 μL of fresh medium was added to each well according to group allocation, and the plates were incubated at 37 °C in a CO_2_ incubator. At the 23rd hour of incubation, the LDH release reagent was added to the maximum enzyme activity control group, and incubation continued until 24 h. All plates were centrifuged at 2,000 rpm for 5 min; then, 120 μL of the supernatant per well was transferred to a new 96-well plate and mixed with 60 μL working solution. After incubation at room temperature for 30 min in the dark, absorbance values at 490 nm and 600 nm were measured. Cytotoxicity was calculated as follows: cytotoxicity (%) = (OD_compound treatment group_ − OD_sample control group_) / (OD_maximum enzyme activity group_ − OD_sample control group_).

### 2.4 Anti-inflammatory effect of GA in an LPS-induced RAW 264.7 cell inflammation model

#### 2.4.1 Establishment of the LPS-induced RAW264.7 cell inflammation model

RAW264.7 cells were seeded onto 12-well plates and incubated for 24 h. The cells were divided into five groups: control group, model (LPS) group, low-dose treatment group (L, 5 μg/mL), medium-dose treatment group (M, 10 μg/mL), and high-dose treatment group (H, 20 μg/mL). After incubation, the medium of the control and model groups was replaced with fresh medium, and the medium of the treatment groups was replaced with fresh medium containing different concentrations of gentisic acid. After 12 h, except for the control group, LPS was added to the cell culture medium of all other groups to induce the inflammatory model. After 6 h, 12 h, and 24 h, the cells and the liquid supernatant of the culture solution were collected for subsequent assays. The level of NO in RAW 264.7 cells was measured using the Griess reagent.

#### 2.4.2 Western blotting analysis

After treatment, cells were harvested for Western blot analysis. In brief, cells were lysed with cold RIPA lysis buffer (containing PMSP, protease inhibitors, and phosphatase inhibitors), and equal amounts of protein were subjected to 12% SDS-PAGE gel electrophoresis and then transferred to PVDF membranes. After 2 h blocking at room temperature in 5% nonfat milk, the membranes were incubated with the primary antibodies at 4 °C overnight, followed by incubation with an appropriate NIR fluorescent secondary antibody for 2 h at room temperature in the dark. The relative densities of the protein bands were quantified using Image Studio software (Pillai-Kastoori et al., 2020).

### 2.5 Anti-inflammatory effect of GA in an OXA-induced zebrafish inflammation model

#### 2.5.1 Establishment of the OXA-induced zebrafish inflammation model

Adult zebrafish were randomly divided into four groups (30 fish per group): a blank control group (CON), a solvent control group (ETOH), a model group (OXA), and a GA treatment group (0.01 mg/kg). The detailed experimental workflow is illustrated in Supplementary Figure S1. The control and model groups were fed a drug-free diet, while the treatment group was given feed containing GA for a continuous period of 14 days, followed by a 12-h fasting period after the last feeding. Subsequently, the zebrafish intestinal oxidative stress model was induced using 0.2% OXA with ethanol as the solvent, as described by Yang et al. (2023a). After the last feeding, the zebrafish were fasted for 24 h and anesthetized with a 0.03% solution of tricaine methanesulfonate. The blank control group was injected with 0.9% physiological saline, the model group and treatment groups were injected with 0.2% OXA, and the solvent control group was injected with 50% ethanol, with an injection volume of 0.6 μL/100 mg for each. After a 24-h fasting period, the zebrafish intestines were collected for subsequent testing.

#### 2.5.2 Histological analysis

Intestinal tissues of zebrafish were collected, cleaned, and fixed in 4% paraformaldehyde. Then, the tissue samples were dehydrated sequentially with gradient ethanol. The tissues were made transparent for paraffin infiltration by replacing the water in the tissues with xylene. The transparent tissues were embedded in paraffin, cooled, and cut into thin sections. After dewaxing the sections, they were stained with hematoxylin–eosin (H&E), dehydrated, mounted, and placed under a microscope to observe and analyze the morphology, arrangement, and structure of the tissues (Cunha and de Brito-Gitirana, 2020; Rehman et al., 2023).

#### 2.5.3 16s rDNA gene sequencing

Bacterial DNA extraction from intestinal contents, PCR amplicon sequencing, and genetic analysis were all performed by Biomarker Technologies Co. Ltd. (Beijing, China). Using the PacBio sequencing platform, the single-molecule real-time sequencing (SMRT Cell) method was performed to sequence the marker genes, followed by circular consensus sequencing (CCS) read filtering to obtain optimized CCS sequences for operational taxonomic unit (OTU) clustering, species annotation, and abundance analysis. Barcoded specific primers were synthesized based on the full-length 16S primers 27F (5′-AGRGTTTGATYNTGGCTCAG-3′) and 1492R (5′-TASGGHTACCTTGTTASGACTT-3′) for PCR amplification. The amplified products were purified, quantified, and homogenized to form a sequencing library (SMRT Bell), which was first subjected to quality inspection and subsequently sequenced using the PacBio Sequel platform. The differences between samples were explored through analyses of α-diversity, β-diversity at the phylum and genus levels, and significant species difference analysis.

#### 2.5.4 Correlation analysis

The microbial diversity analysis was performed using the Biomarker Technologies Online Platform (https://www.biocloud.net/). The correlation analysis was performed using Pearson rank correlation analysis, and the significances were calculated.

### 2.6 Quantitative real-time PCR (qPCR)

Total RNA from RAW 264.7 cells and intestinal tissues of zebrafish was isolated using TRIzol reagent, and the extraction process was strictly carried out in accordance with the manufacturer’s instructions. After RNA extraction, the mRNA was reverse-transcribed into cDNA using TransScript® First-Strand cDNA Synthesis SuperMix, following the standard protocol provided by the manufacturer. The obtained cDNA was used as the template for qRT-PCR reactions, which were performed using the SYBR green chemistry detection method (Pérez-Osorio and Franklin, 2008). The specific primer sequences used in the qPCR analysis for RAW 264.7 cells and zebrafish are listed in Table 1, 2, respectively.

**TABLE 1:** Primer sequence (RAW 264.7 cells).

Gene	Sequences (forward)	Sequences (reverse)
GAPDH	F- GTA​ACC​CGT​TGA​ACC​CCA​TT	R- CCA​TCC​AAT​CGG​TAG​TAG​CG
IL-1β	F-GCAGCAGCACATCAACAAGAGC	R-AGGTCCACGGGAAAGACACAGG
TNF-α	F- GTG​CCA​GCC​GAT​GGG​TTG​TAC	R- TGA​CGG​CAG​AGA​GGA​GGT​TGA​C
IL-6	F- GCC​TTC​TTG​GGA​CTG​ATG​CT	R- GGT​CTG​TTG​GGA​GTG​GTA​TCC
TRAF6	F- ATA​TGA​CAG​CCA​CCT​CCC​CT	R- GGC​AAG​CAG​TTC​TGG​TTT​GG
IL-17A	F- TAC​CTC​AAC​CGT​TCC​ACG​TC	R- TCA​GGG​TCT​TCA​TTG​CGG​TG
IKKα	F- GCA​GAC​CGT​GAA​CAT​CCT​CT	R- TCC​AGG​ACA​GTG​AAC​GAG​TG
IKKβ	F- AGG​CGA​CAC​GTG​AAC​AGA​T	R- CTAAGAGCGGATGCGATG
TNFR1	F- GCT​GTT​GCC​CCT​GGT​TAT​CT	R- ATG​GAG​TAG​ACT​TCG​GGC​CT
TRAF2	F- CCT​CAG​GTG​TGC​ATC​CAT​TCT	R- TCG​TGG​CAG​CTC​TCG​TAT​TC

**TABLE 2:** Primer sequences (Zebrafish).

Gene	Sequences (forward)	Sequences (reverse)
GAPDH	F- TGC​TGG​TAT​TGC​TCT​CAA​CG	R- GCC​ATC​AGG​TCA​CAT​ACA​CG
IL-6	F- TCA​ACT​TCT​CCA​GCG​TGA​TG	R- TCT​TTC​CCT​CTT​TTC​CTC​CTG
IL-1β	F- GGC​TGT​GTG​TTT​GGG​AAT​CT	R- TGA​TAA​ACC​AAC​CGG​GAC​A
IL-10	F- ATT​TGT​GGA​GGG​CTT​TCC​TT	R- AGA​GCT​GTT​GGC​AGA​ATG​GT
MUC2	F- CAA​CAT​CGA​TGG​CTG​CTT​CTG	R- CTG​ACA​GTA​ACA​TTC​TTC​CTC​GC
ZO-1	F- CAG​GGC​GTC​AAG​AAC​ATG​AGG	R- GTG​GTG​GTG​AAA​AGG​TGA​TGG
Occludin	F- AGC​CGG​CGT​ACT​CCT​ACT​AT	R- GGA​GGC​CAC​ACA​GAC​AAA​GA
IKKα	F- TAA​CGG​CAC​ACT​GTC​AAA​GC	R- GTG​GCT​CTC​TGC​TCC​TGG​T
IκBα	F- GGT​GGA​AAG​ACT​CCT​GAA​AGC	R- TGT​AGT​TAG​GGA​AGG​TAA​GAA​TG
P65	F- GCA​AGA​TGA​GAA​CGG​AGA​CAC	R- CTA​CCA​GCA​ATC​GCA​AAC​AA
TRAF6	F- TGC​GCT​TTC​TGA​CCA​TCT​GT	R- TCA​GCA​CAG​CAG​ATT​AGG​GC
IL-17A	F- CCA​CGA​TGG​AGT​TAC​CAG​CTA	R- TGT​GTC​TGT​GTG​AGT​TTG​AGT​GTT

### 2.7 Statistical analysis

The experimental data were statistically analyzed, and graphs were generated using GraphPad Prism 8.0 software, while experimental image analysis was conducted using ImageJ software. All experimental results are presented as the mean ± standard deviation (SD). Statistical analyses were performed using one-way analysis of variance (ANOVA) followed by the least significance difference (LSD) test when more than two groups were compared. A *P*-value of <0.05 was considered statistically significant. All experiments were performed with n = 3 independent biological replicates to ensure the reliability and reproducibility of the experimental results.

## 3 Results

### 3.1 Network pharmacology analysis of anti-inflammatory targets and pathways of GA

**FIGURE 1:**
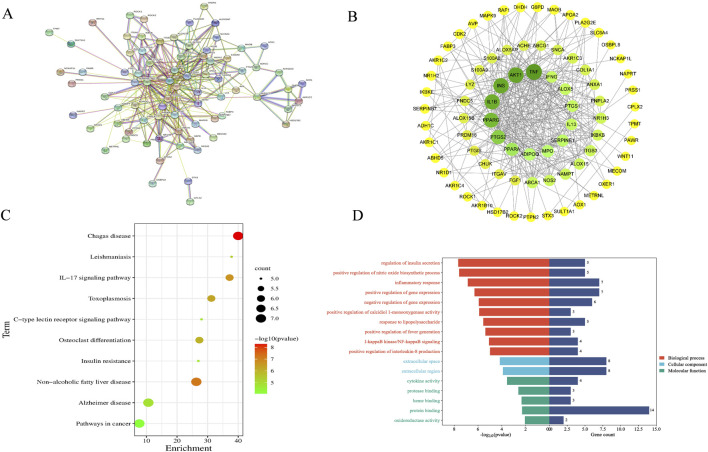
Network analysis of the anti-inflammatory effect of GA. **(A)** PPI network of anti-inflammatory targets. **(B)** PPI network visualized using Cytoscape. **(C)** KEGG enrichment analysis results. **(D)** GO enrichment analysis results.

The PPI network analysis identified a total of 83 targets and 352 interaction edges (Figure 1A). The network was further visualized using Cytoscape software, as presented in Figure 1B. Targets with a degree value greater than 10 were screened as the core targets of GA. Among them, the top five targets ranked by degree value were TNF, AKT1, INS, IL1B, and PPARG, as listed in Table 3. The KEGG analysis results showed that the pathway directly related to inflammation by GA was the IL-17 signaling pathway (Figure 1C). The GO analysis results showed that the targets of GA were mainly involved in the regulation of insulin secretion, positive regulation of the nitric oxide biosynthetic process, inflammatory response, and other biological processes. Moreover, these targets primarily regulated biological processes in locations such as the extracellular space and extracellular region, through mechanisms such as cytokine activity (Figure 1D). Therefore, in the subsequent experiments, we examined the expression of the key targets in the IL-17 signaling pathway at the gene and protein levels.

**TABLE 3:** Core targets of GA for anti-inflammatory.

No.	Protein name	Gene symbol	Degree
1	Tumor necrosis factor	TNF	39
2	RAC-alpha serine/threonine-protein kinase	AKT1	37
3	Insulin [cleaved into: insulin B chain and insulin A chain]	INS	36
4	Interleukin-1 beta	IL1B	35
5	Peroxisome proliferator-activated receptor gamma	PPARG	33
6	Prostaglandin G/H synthase 2	PTGS2	29
7	Peroxisome proliferator-activated receptor alpha	PPARA	24
8	Myeloperoxidase	MPO	19
9	Adiponectin	ADIPOQ	19
10	Plasminogen activator inhibitor 1	SERPINE1	17
11	Interferon gamma	IFNG	15
12	Interleukin-13	IL13	15
13	Prostaglandin G/H synthase 1	PTGS1	15
14	Polyunsaturated fatty acid 5-lipoxygenase	ALOX5	15
15	Phospholipid-transporting ATPase ABCA1	ABCA1	14
16	Nicotinamide phosphoribosyltransferase	NAMPT	13
17	Nitric oxide synthase, inducible	NOS2	13
18	Polyunsaturated fatty acid lipoxygenase ALOX15	ALOX15	13
19	Integrin beta-3	ITGB3	13
20	Oxysterol receptor LXR-alpha	NR1H3	12
21	Inhibitor of nuclear factor kappa-B kinase subunit beta	IKBKB	12
22	Patatin-like phospholipase domain-containing protein 2	PNPLA2	10
23	Aldo-keto reductase family 1 member C3	AKR1C3	10
24	Collagen alpha-1	COL1A1	10
25	Annexin A1	ANXA1	10

### 3.2 Effects of GA treatment on cell viability

**FIGURE 2:**
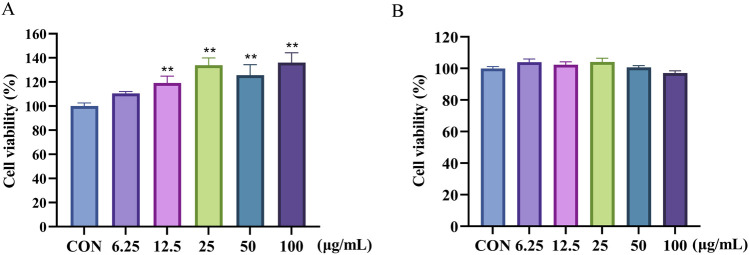
Effects of GA treatment on cell viability in RAW 264.7 cells. **(A)** Cell viability was measured using the CCK8 assay. **(B)** Cell viability was measured using the LDH release assay. The values are expressed as the mean ± SD (n = 3 per group). All data were analyzed using one-way ANOVA followed by the LSD test (compared with the control group, ^**^ *P* < 0.01). The cell viability of the control group was normalized to 100%.

To evaluate the cell viability of GA to RAW 264.7 cells, the CCK8 assay and LDH release assay were performed using different concentrations of GA (6.25, 12.5, 25, 50, and 100 μg/mL) for the treatment of RAW 264.7 cells for 24 h. As shown in Figures 2A,B, the results showed that GA did not produce significant toxic effects on RAW264.7 cells but rather promoted cell growth.

### 3.3 Anti-inflammatory effect of GA in an LPS-induced RAW 264.7 cells inflammation model

#### 3.3.1 Effects of GA on inflammatory response in RAW 264.7 cells

**FIGURE 3:**
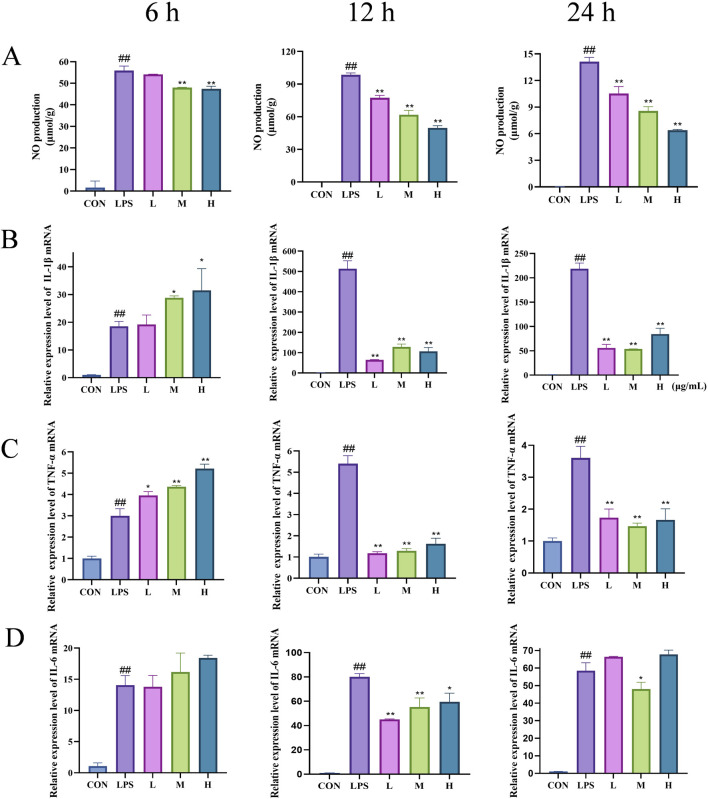
Effects of GA on the level of NO and inflammatory factors in RAW264.7 cells. **(A)** NO production. **(B)** Relative expression level of IL-1β mRNA. **(C)** Relative expression level of TNF-α mRNA. **(D)** Relative expression level of IL-6 mRNA. Cells were pretreated with GA at low dose (L, 5 μg/mL), medium dose (M, 10 μg/mL), and high dose (H, 20 μg/mL), followed by LPS stimulation. The values are expressed as the mean ± SD (n = 3 per group). All data were analyzed using one-way ANOVA followed by the LSD test (compared with the control group, ^#^ *P* < 0.05 and ^##^ *P* < 0.01; compared with the model group, ^*^ *P* < 0.05 and ^**^ *P* < 0.01).

Upon stimulation with LPS, RAW 264.7 cells secrete substantial amounts of NO and pro-inflammatory cytokines, which serve as robust surrogate markers for the magnitude of the inflammatory response. Consequently, the anti-inflammatory efficacy of GA can be rapidly assessed by quantifying these mediators. As depicted in Figures 3A–D, LPS challenge significantly elevated NO production and the mRNA transcript levels of IL-1β, TNF-α, and IL-6 in RAW 264.7 cells (*P* < 0.01), thereby validating the successful establishment of the LPS-induced inflammatory model. Pretreatment with GA potently inhibited LPS-driven inflammatory responses, with its anti-inflammatory activity displaying marked time dependency and target specificity. Specifically, the inhibition of NO release exhibited a clear dose-dependent reduction across all pretreatment time points (6 h, 12 h, and 24 h), with the most pronounced suppression observed at the highest concentration (20 μg/mL) and following 24 h of pretreatment (*P* < 0.01). In contrast, attenuation of IL-1β, TNF-α, and IL-6 mRNA expression was only evident in 12 h and 24 h of pretreatment (*P* < 0.01), with no discernible dose-dependent effect. In addition, combined with the target characteristics screened from the PPI network, TNF and IL1B were identified as core inflammatory hub targets; their mRNA levels were most significantly downregulated at 12 h of GA pretreatment, which greatly ameliorated LPS-mediated inflammatory response. Accordingly, 12 h was ultimately determined as the optimal intervention time for subsequent exploration of the regulatory effects of GA on key targets and signaling pathways in RAW264.7 cells. This observation implies that GA-mediated regulation of pro-inflammatory cytokine transcription is primarily governed by pretreatment duration rather than concentration. Notably, 6 h of pretreatment elicited only a marginal inhibitory trend in cytokine expression in select high-concentration groups, further corroborating that the anti-inflammatory potential of GA requires a sufficient duration of exposure.

#### 3.3.2 Effects of GA on the key targets and pathway in RAW264.7 cells

**FIGURE 4:**
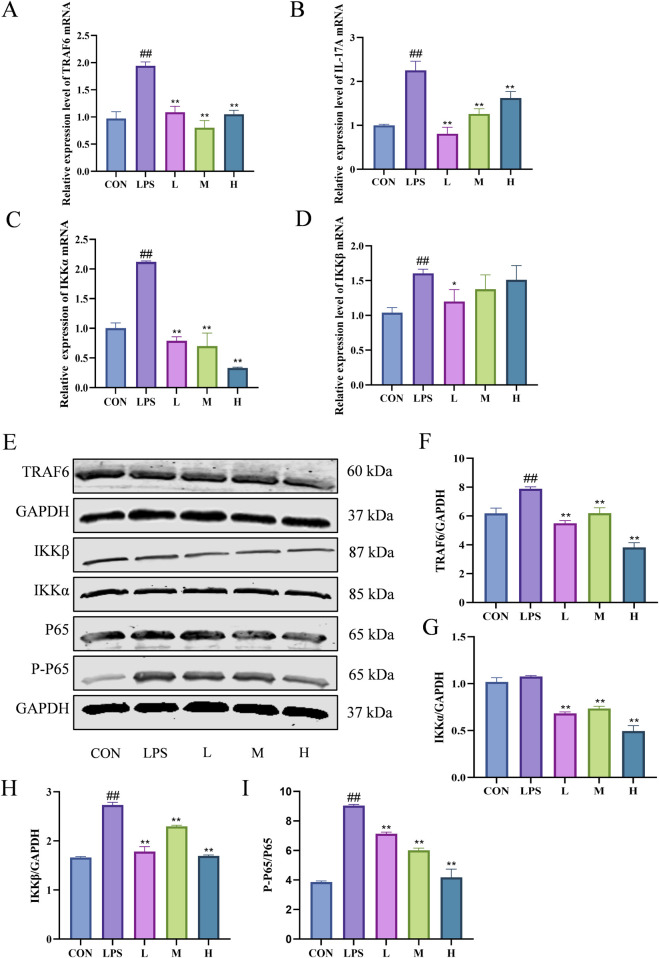
The effect of GA on mRNA and proteins levels of key targets in the IL-17 signaling pathway. **(A)** The expression level of *TRAF6* mRNA. **(B)** The expression level of *IL-17A* mRNA. **(C)** The expression level of *IKKα* mRNA. **(D)** The expression level of *IKKβ* mRNA. **(E)** Western blot analysis of key target proteins. **(F)** The protein expression level of TRAF6. **(G)** The protein expression level of IKKα. **(H)** The protein expression level of IKKβ. **(I)** The protein expression level of p-p65. Cells were pretreated with GA at low dose (L, 5 μg/mL), medium dose (M, 10 μg/mL), and high dose (H, 20 μg/mL), followed by LPS stimulation. The values are expressed as the mean ± SD (n = 3 per group). All data were analyzed using one-way ANOVA followed by the LSD test (compared with the control group, ^#^ *P* < 0.05 and ^##^ *P* < 0.01; compared with the model group, ^*^ *P* < 0.05 and ^**^ *P* < 0.01).

As shown in Figures 4A–I, both qRT-PCR and Western blotting results demonstrated consistent changes in the IL-17 signaling pathway-related molecules. LPS stimulation markedly upregulated the mRNA relative expression of *TRAF6*, *IL-17A*, *IKKα*, and *IKKβ*, as well as the protein levels of TRAF6 and IKKβ and the phosphorylation level of p65 (*P* < 0.01). Intervention with GA obviously reversed these LPS-induced upregulations and significantly downregulated both the mRNA transcription and protein expression of the above key molecules in the IL-17 pathway. Collectively, these findings indicated that GA exerts its anti-inflammatory activity by suppressing the activation of the IL-17 signaling pathway in RAW264.7 cells.

### 3.4 Anti-inflammatory effect of GA in an OXA-induced zebrafish inflammation model

#### 3.4.1 Effects of GA on intestinal tissue morphology of zebrafish

**FIGURE 5:**
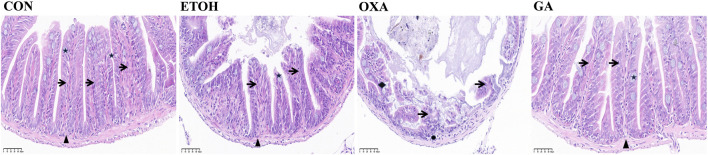
Effects of GA on the intestinal tissue of zebrafish. Black triangles represent the muscular layer, black arrows represent mucosal villi, black five-pointed stars represent epithelial goblet cells, black diamonds represent vacuolar degeneration, and black circles represent inflammatory cell infiltration.

As shown in Figure 5, the intestinal tissue structure of zebrafish in the control group was normal, with visible mucosa, submucosa, and muscular layer. The mucosal villi were neatly arranged, and goblet cells in the epithelium were scattered. No degeneration or necrosis was observed, and there was no inflammatory cell infiltration in the lamina propria or other pathological changes. Compared with the control group, the intestinal tissue structure of zebrafish in the EtOH solvent group was normal, with no significant pathological changes observed. After exposure to OXA, the intestinal villi of zebrafish were atrophied and shortened, with disordered arrangement. Vacuolar degeneration was observed in the epithelial cells, and inflammatory cell infiltration was present in the lamina propria. After administration of GA (0.01 mg/kg), the intestinal tissue structure of zebrafish returned to normal, with the inflammatory-related changes subsiding and no significant pathological changes observed.

#### 3.4.2 Effects of GA on key genes in zebrafish

**FIGURE 6:**
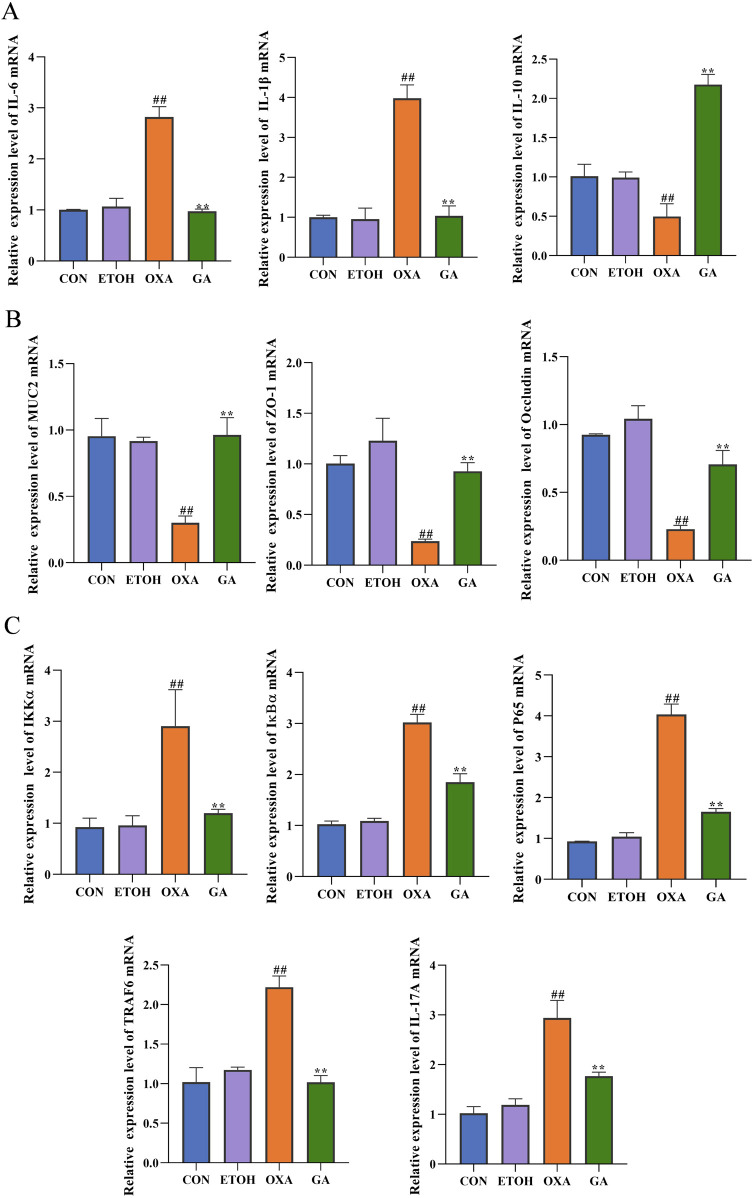
Effect of GA on mRNA expression of key genes in zebrafish. **(A)** The expression level of inflammatory factor-related genes. **(B)** The expression level of intestinal barrier-related genes. **(C)** The expression level of key genes in the IL-17 signaling pathway. The values are expressed as the mean ± SD (n = 3 per group). All data were analyzed using one-way ANOVA followed by the LSD test (compared with the control group, ^#^ *P* < 0.05 and ^##^ *P* < 0.01; compared with the model group, ^*^ *P* < 0.05 and ^**^ *P* < 0.01).

As depicted in Figures 6A–C, there was no significant difference between the EtOH group and the control group, indicating that ethanol has no significant impact on the experimental results and could be used as a solvent for OXA. As shown in Figure 6A, compared with the control group, OXA significantly increased the expression levels of inflammatory cytokines *IL-6* mRNA and *IL-1β* mRNA and significantly decreased the expression level of the anti-inflammatory cytokine *IL-10* mRNA (*P* < 0.01), indicating the successful establishment of OXA-induced intestinal inflammation in adult zebrafish. Compared with the model group, GA (0.01 mg/kg) could significantly reduce the expression levels of the inflammatory cytokines *IL-6* mRNA and *IL-1β* mRNA and significantly increased the expression level of the anti-inflammatory cytokine *IL-10* mRNA (*P* < 0.01), demonstrating that GA had significant anti-inflammatory effects.

As shown in Figure 6B, compared with the control group, OXA significantly reduced the expression levels of the intestinal barrier-related proteins *MUC2* mRNA, *ZO-1* mRNA, and *Occludin* mRNA, indicating that an appropriate amount of OXA could significantly damage the intestinal barrier. Compared with the model group, GA significantly increased the expression levels of *MUC2* mRNA, *ZO-1* mRNA, and *Occludin* mRNA (*P* < 0.01), indicating that GA could significantly improve intestinal barrier and repair intestinal damage.

As shown in Figure 6C, OXA significantly increased the expression levels of *IKKa* mRNA, *IκBα* mRNA, *TRAF6* mRNA, *p65* mRNA, and *IL-17A* mRNA. Compared with the model group, GA was able to significantly inhibit the expression levels of *IKKa mRNA*, *IκBα mRNA*, *TRAF6 mRNA*, *p65 mRNA*, and *IL-17A* mRNA (*P* < 0.01), suggesting that GA may exert its anti-inflammatory effects through the IL-17A signaling pathway and contribute to the repair of intestinal damage.

#### 3.4.3 Effects of GA on intestinal microbiota in zebrafish

**FIGURE 7:**
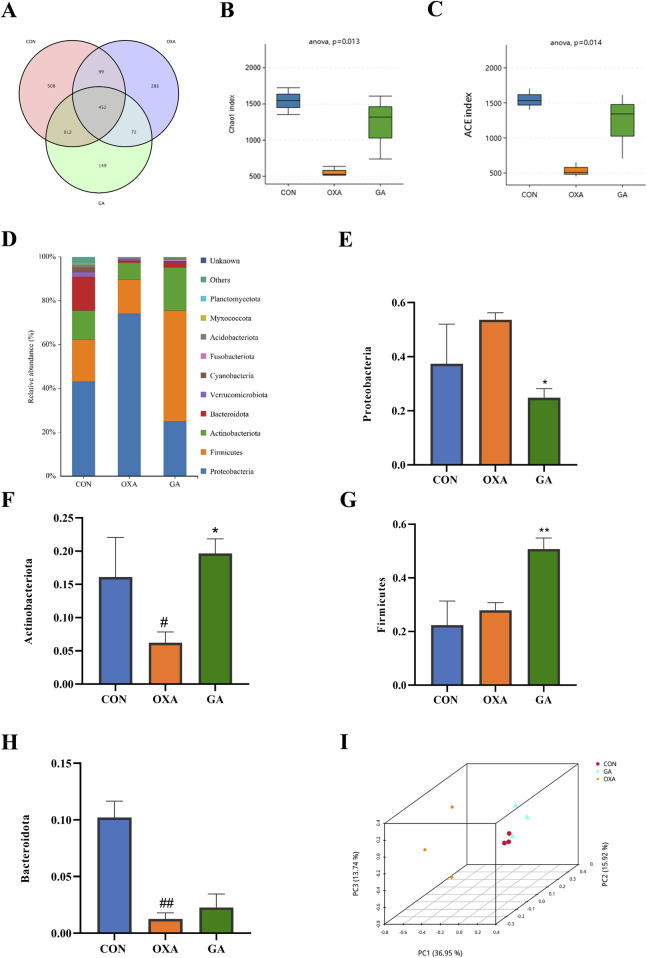
Effects of GA on intestinal microbiota in zebrafish. **(A)** A Venn diagram shows the overlap of the OTUs identified in the intestinal microbiota among CON, OXA, and GA groups. **(B)** Analysis of alpha diversity (Chao1 index). **(C)** Analysis of alpha diversity (ACE index). **(D)** The top 10 bacteria, with a maximum abundance of gut bacteria at the phylum level. **(E)** Abundance of *Proteobacteria*. **(F)** Abundance of *Actinobacteriota*. **(G)** Abundance of *Firmicutes*. **(H)** Abundance of *Bacteroidota*. **(I)** Plots of unweighted UniFrac-based PCoA. The values are expressed as the mean ± SD (n = 3 per group). All data were analyzed using one-way ANOVA followed by the LSD test (compared with the control group, ^#^ *P* < 0.05 and ^##^ *P* < 0.01; compared with the model group, ^*^ *P* < 0.05 and ^**^ *P* < 0.01).

Significant changes in the gut microbiota composition were observed in zebrafish in the OXA-induced model group, and these alterations were partially restored by GA treatment (Figure 7). The unique OTU counts for the control, model, and treatment groups were 508, 281, and 149, respectively (Figure 7A). Compared with the control group, the Chao1 and ACE indices in the model group decreased, and the Chao1 and ACE indices in the treatment group significantly increased (*P* < 0.05), indicating that exposure to OXA reduced the community diversity of the zebrafish gut microbiota, while GA enhanced the diversity of the zebrafish gut microbiota (Figures 7B,C).

Further analysis of species abundance found that, at the phylum level, *Proteobacteria*, *Firmicutes*, and *Actinobacteriota* were among the most abundant taxa, and OXA significantly reduced the abundance of *Actinobacteriota* and *Bacteroidota*. GA significantly increased the abundance of *Actinobacteriota* (Figures 7D,F,H). PCoA analysis further indicated that there was a clear separation of gut microbiota among the three groups at the phylum level (Figure 7I).

#### 3.4.4 Effects of the association between the biomarkers of key genes and intestinal microbiota in zebrafish

**FIGURE 8:**
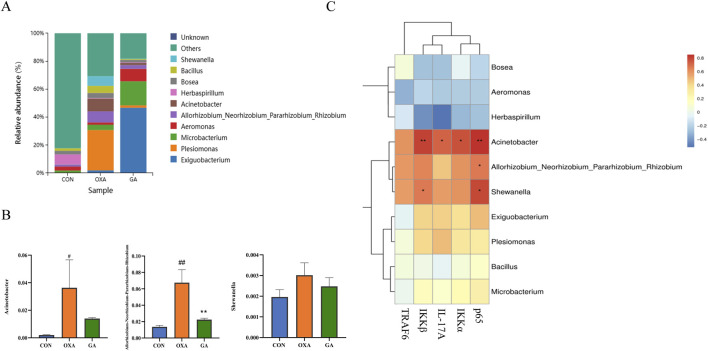
Effects of the association between the biomarkers of key genes and intestinal microbiota in zebrafish. **(A)** The top 10 bacteria, with a maximum abundance of gut bacteria at the genus level. **(B)** Abundance of *Acinetobacter*, *Allorhizobium–Neorhizobium–Pararhizobium–Rhizobium*, and *Shewanella*. The values are expressed as the mean ± SD (n = 3 per group). All data were analyzed using one-way ANOVA followed by the LSD test (compared with the control group, ^#^ *P* < 0.05 and ^##^ *P* < 0.01; compared with the model group, ^*^ *P* < 0.05 and ^**^ *P* < 0.01). **(C)** The Pearson analysis between key genes in the IL-17 signaling pathway and intestinal microbiota at the genus level. The rows represent species, and the columns represent environmental factors. The color of the blocks indicates the magnitude of the Pearson correlation coefficient, with ^*^ *P* < 0.05 and ^**^ *P* < 0.01.

Further analysis of the abundance proportions of the top 10 genera (Figures 8A,B) demonstrated that OXA treatment altered the native structure of the zebrafish intestinal microbiota and significantly increased the relative abundance of *Acinetobacter* and *Allorhizobium–Neorhizobium–Pararhizobium–Rhizobium* (*P* < 0.01 and *P* < 0.05). Interventions with GA caused a significant reduction in abundance of *Allorhizobium–Neorhizobium–Pararhizobium–Rhizobium* (*P* < 0.01).

As shown in Figure 8C, the relationship between the oxidative stress-related biomarkers and the abundance of the top 10 microbial species were studied by calculating the Pearson correlation coefficient; the correlation and significance *P*-values between each pair were obtained. *Acinetobacter* was positively correlated with the changes in *IKKβ*, *IL-17A*, *IKKα*, and *p65* content; *Allorhizobium–Neorhizobium–Pararhizobium–Rhizobium* was positively correlated with *p65*; *Shewanella* was positively correlated with *IKKβ* and *p65*.

## 4 Discussion

GA, a benzoic acid derivative present in various food raw materials, has been proven to exert multiple pharmacological activities, including anti-cancer and antioxidant effects (Kang et al., 2021). It is an important metabolite of aspirin and also widely exists in plants as a plant secondary metabolite (Fernández et al., 2010; Hosseinzadeh et al., 2025).

According to the pathway enrichment results of network pharmacology analysis, GA exerts its anti-inflammatory effect primarily through the IL-17 signaling pathway. Studies have reported that the IL-17 signaling pathway functions by inducing the secretion of downstream cytokines and mainly mediates inflammatory responses and hematopoietic functions (Berry et al., 2022; Zhang and Shen, 2025). Upon stimulation, interleukin-17 receptor A (IL-17RA) first binds to IL-17A and then recruits the adaptor protein Act1, which mediates the expression of TRAF6, followed by the activation of the downstream NF-κB pathway and mitogen-activated protein kinase (MAPK) signaling pathway (Swaidani et al., 2019; Yang et al., 2023b).

In this study, the dosages of GA for *in vitro* RAW264.7 cell experiments and *in vivo* zebrafish intervention were rationally determined. We referenced reported effective concentrations of GA (1, 5, and 10 μg/mL) in HaCaT cells affecting keratinocyte proliferation and re-epithelization (Mentese et al., 2023). Our CCK-8 results further verified that GA had no obvious cytotoxicity against RAW264.7 cells even at 100 μg/mL. Accordingly, 5, 10, and 20 μg/mL were selected as low, medium, and high doses, respectively, for subsequent NO detection and qPCR analysis to explore the dose-dependent anti-inflammatory effect of GA. For the *in vivo* dose, the natural content of GA in plants is generally 0.1–40 mg/100 g dry weight (Rababah et al., 2011). Diet supplementation with 0.1% GA-containing plant materials yields a theoretical physiological concentration of 0.01 mg/kg, consistent with the natural background exposure of aquatic organisms. Moreover, our previous work demonstrated that similar phenolic acids exerted prominent intestinal protective effects in zebrafish at comparable low doses (Zhou et al., 2026). Therefore, 0.01 mg/kg was adopted as the dietary intervention dose for subsequent phenotypic and mechanistic analyses in zebrafish.

*In vitro* experiments on RAW264.7 cells demonstrated that GA could significantly reduce the expressions of LPS-induced inflammatory mediator NO and pro-inflammatory cytokines including TNF-α, IL-6, and IL-1β, with the optimal anti-inflammatory effect observed at 12 h of pretreatment (Bian et al., 2019). The *in vitro* anti-inflammatory activity is mainly achieved by blocking the IL-17 signaling pathway. GA remarkably downregulated the mRNA and protein expression levels of *TRAF6*, *IL-17A*, and inhibitors of *IKKα* and *IKKβ* in this pathway and inhibited the phosphorylation of NF-κB/p65, thereby blocking the activation of the downstream NF-κB signaling pathway. In the present study, pretreatment with GA significantly decreased the mRNA and protein expression levels of TRAF6, IL-17A, IKKα, and IKKβ and also reduced the phosphorylation level of NF-κB/p65 in LPS-induced mouse mononuclear macrophage RAW264.7 cells. These findings further verified the network prediction results that GA could inhibit the activation of the NF-κB signaling pathway by blocking the IL-17 signaling pathway, thus exerting an anti-inflammatory effect.

OXA disrupts the balance between the gut microbiota and intestinal immune system, and alterations in gut microbial composition are closely associated with the balance of pro-inflammatory and anti-inflammatory responses in the intestine (Zhao et al., 2022; Desai et al., 2016; Di Vincenzo et al., 2024; Quaglio et al., 2022). Extensive studies have revealed that gut dysbiosis and associated inflammation are linked to a wide range of diseases, including diabetes, arthritis, systemic lupus erythematosus, cancer, and cardiometabolic disorders (Nesci et al., 2023; Erridge, 2011; Bagheri et al., 2022; Zhao et al., 2021; Mou et al., 2022). In previous studies, we have successfully established an OXA-induced zebrafish inflammation model and conducted investigations on the anti-inflammatory activity of natural plant extracts based on this model. It has been reported that the gut microbiota of normal adult zebrafish is mainly composed of the phyla *Proteobacteria*, *Firmicutes*, *Actinobacteriota*, and *Bacteroidota* (Sun et al., 2019; Kang et al., 2021), and the sequencing results of the present study also confirmed this microbial composition characteristic. Notably, GA intervention alone could significantly reverse the OXA-induced reduction in *Actinobacteriota*. Further analysis of microbial abundance at the genus level confirmed that GA intervention decreased the abundances of *Acinetobacter*, *Allorhizobium–Neorhizobium–Pararhizobium–Rhizobium*, and *Shewanella*. Studies have indicated that the pathogenic bacterium *Acinetobacter* proliferates significantly during intestinal inflammation and can produce endotoxins to induce inflammatory responses, thus being regarded as one of the core pro-inflammatory bacteria (Doughari et al., 2011; Fan et al., 2025; Scribano et al., 2025). *Allorhizobium–Neorhizobium–Pararhizobium–Rhizobium* is a common commensal bacterium in the zebrafish gut with relatively stable abundance under normal physiological conditions, and its abundance changes significantly (either increase or decrease) when exposed to environmental stress (Bunyoo et al., 2022; Zhang et al., 2022). *Shewanella*, a bacterium derived from aquatic environments belonging to the *Gammaproteobacteria* class, is a transient bacterium with extremely low abundance in the zebrafish gut that is almost undetectable under normal conditions. It proliferates specifically during intestinal inflammation, especially oxidative stress-induced inflammation (Imperatore et al., 2022; Paździor et al., 2023). As *Acinetobacter* is a core pro-inflammatory bacterium in intestinal inflammation and the latter two genera proliferate specifically during the occurrence of intestinal inflammation, the above results confirmed that GA was capable of modulating intestinal microecological structure and was associated with decreased relative abundances of *Acinetobacter*, *Allorhizobium–Neorhizobium–Pararhizobium–Rhizobium*, and *Shewanella* in the zebrafish gut.

Pearson correlation analysis further confirmed that the three bacterial strains inhibited by GA were significantly positively correlated with the key molecules of the IL-17/NF-κB signaling pathway. *Acinetobacter* was positively correlated with the expression changes of IKKβ, IL-17A, IKKα, and p65. *Allorhizobium–Neorhizobium–Pararhizobium–Rhizobium* was positively correlated with p65, and *Shewanella* was positively correlated with IKKβ and p65. Taken together, these results were consistent with the downregulatory effect of GA on the key genes/proteins of the IL-17 signaling pathway, its upregulatory effect on intestinal tight junction proteins, and its correlated regulatory trend on the gut microbiota.

**FIGURE 9:**
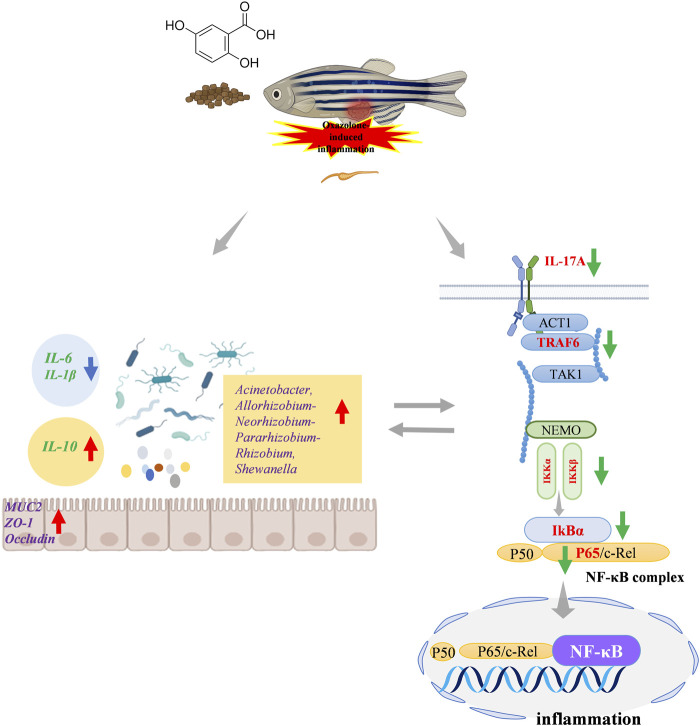
The anti-inflammatory mechanisms of GA. (Created in BioRender. Weiwei, Z. (2026) https://BioRender.com/08qu6vo).

The potential dual regulatory patterns underlying the intestinal anti-inflammatory action of GA were preliminarily elucidated. At the intestinal microenvironmental level, GA is capable of regulating intestinal microecological homeostasis, which is accompanied by reduced relative abundance of abnormally proliferative *Acinetobacter*, *Allorhizobium–Neorhizobium–Pararhizobium–Rhizobium*, and *Shewanella* in zebrafish intestines. At the molecular signaling level, GA may suppress excessive activation of the IL-17/NF-κB pathway, facilitate intestinal mucosal barrier restoration, and thereby alleviate inflammatory responses. These two interrelated regulatory modes are speculated to exert synergistic effects and potentially mediate the intestinal anti-inflammatory activity of GA (Figure 9). These multiple pathways synergistically exert a protective effect against intestinal inflammation. In addition, GA has the potential to be developed as a therapeutic drug or a feed additive for improving intestinal health in animals.

Although this study systematically explored the anti-inflammatory activity and correlative regulatory characteristics of GA and verified its effects in *in vitro* cell and zebrafish *in vivo* models, it had certain limitations. This study only used zebrafish as the *in vivo* model, and the research results required further verification in other mammalian models. Notably, pathway verification in zebrafish was mainly performed at the mRNA level, and corresponding confirmation at the protein level in zebrafish tissues was not conducted, which should be supplemented in subsequent experiments. Meanwhile, the exploration of intestinal flora regulation mechanism was merely limited to at the level of abundance and correlation analysis, and definite causal relationships, as well as key functional metabolites, were not identified. In addition, the study on anti-inflammatory mechanism focused on the IL-17/NF-κB pathway, lacking analysis of molecular binding ability and *in vivo* tissue-level verification. In the future, targeted studies will be conducted, including constructing mammalian inflammation models, performing fecal microbiota transplantation and metabolomics analysis, exploring multi-target mechanisms and tissue-level verification, and investigating its synergistic effect with other natural compounds to tap its industrial application potential.

## 5 Conclusion

In summary, GA, as a typical polyphenolic compound with potential anti-inflammatory activity, may exert its anti-inflammatory effects by coordinately regulating the composition of pathogenic intestinal bacteria and modulating the IL-17/NF-κB signaling pathway. Specifically, GA can participate in the regulation of the community structure of pathogenic intestinal bacteria (*Acinetobacter*, *Allorhizobium–Neorhizobium–Pararhizobium–Rhizobium*, and *Shewanella*), thereby maintaining intestinal microecological homeostasis, which is a key factor in alleviating intestinal inflammation. Meanwhile, GA can also coordinately regulate the IL-17/NF-κB signaling pathway, which plays a crucial role in the regulation of intestinal inflammatory responses. Taken together, the present study systematically elucidates the multi-pathway anti-inflammatory molecular mechanism of GA, which lays a solid theoretical foundation for the in-depth development and clinical application of polyphenol-derived bioactive substances.

## Data Availability

The data presented in the study are deposited in the NCBI repository, accession number PRJNA1478867.
