# Supplementary material for: Study on the anti-inflammatory effects and mechanisms of gentisic acid based on the LPS-induced RAW264.7 cell inflammation model and the oxazolone-induced zebrafish inflammation model
Source: Front Pharmacol. 2026 Jun 26;17:1837686. doi: 10.3389/fphar.2026.1837686 (PMC13350250; doi:10.3389/fphar.2026.1837686)
Supplement: Supplementary file 1 [file Supplementaryfile1.docx]

Figure 1. Original western blot


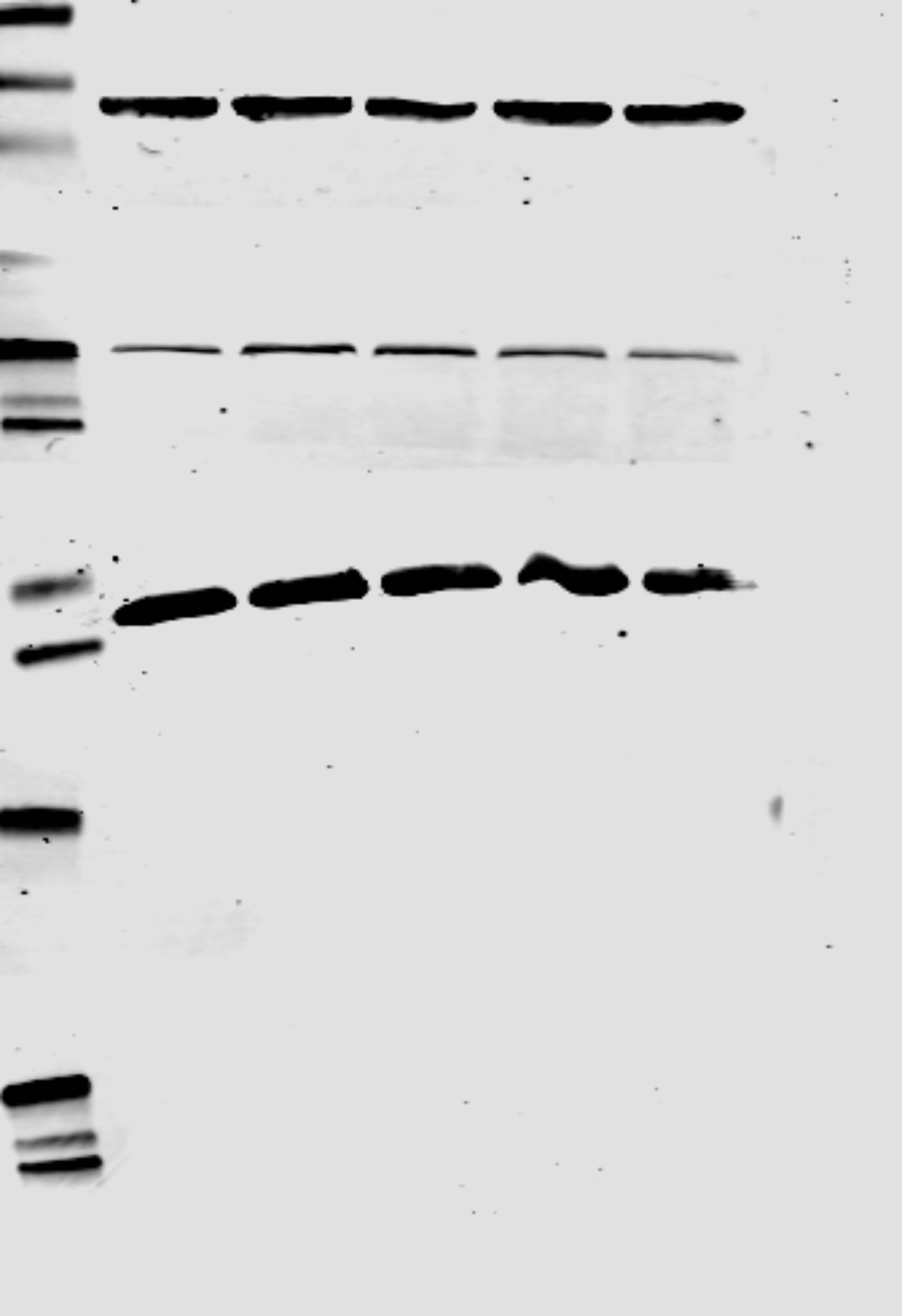


180

IKKβ 87 kDa 130


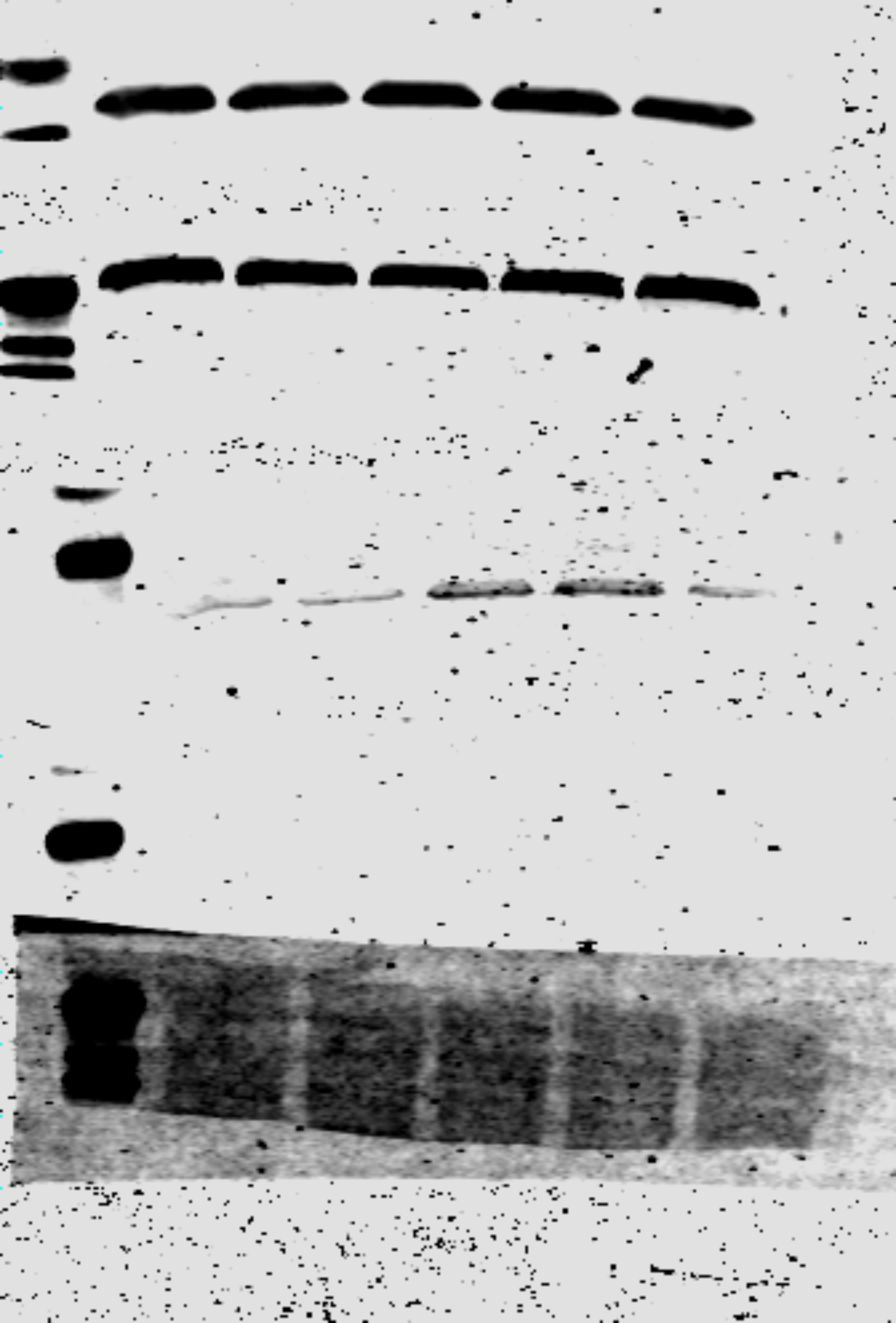
 100

180

IKKα 85 kDa 130

100


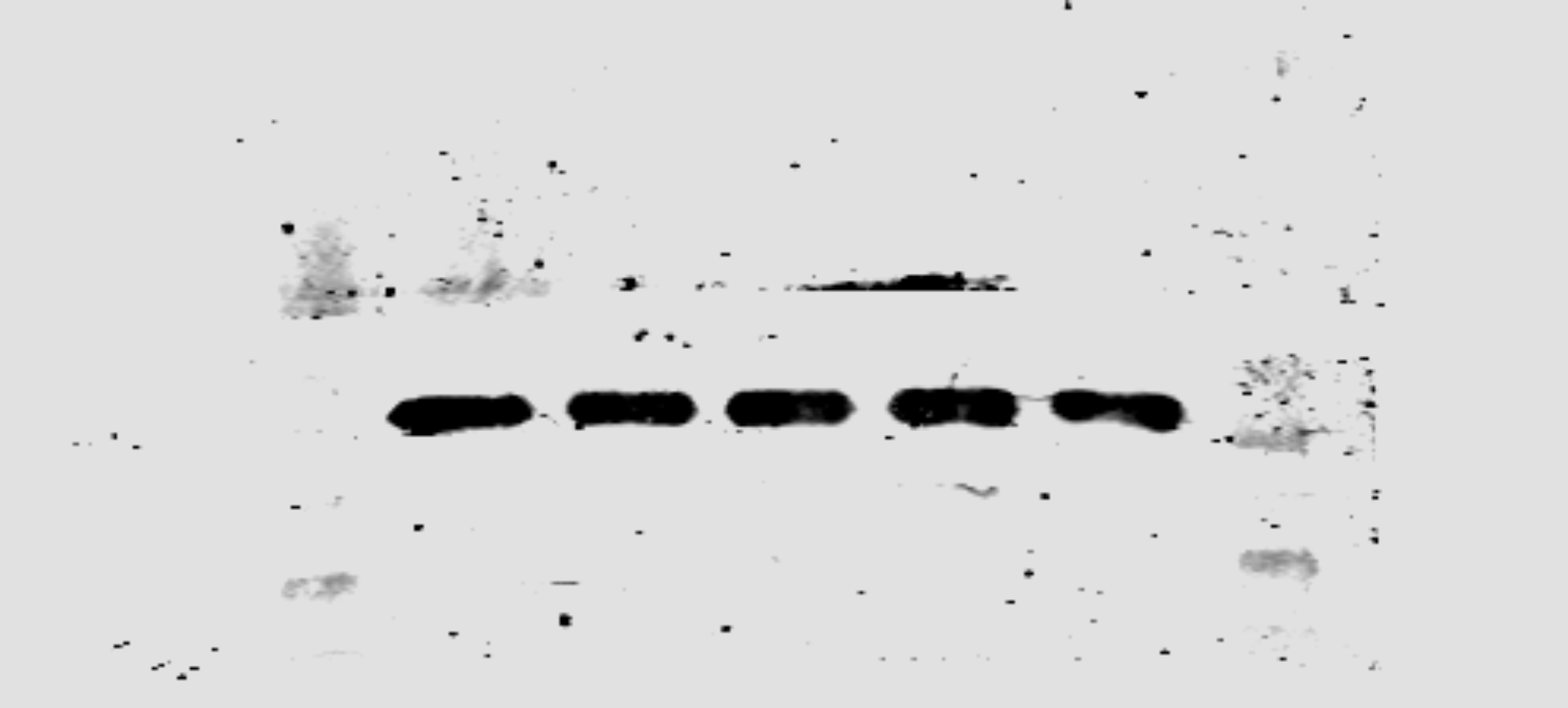


P65 65 kDa

55


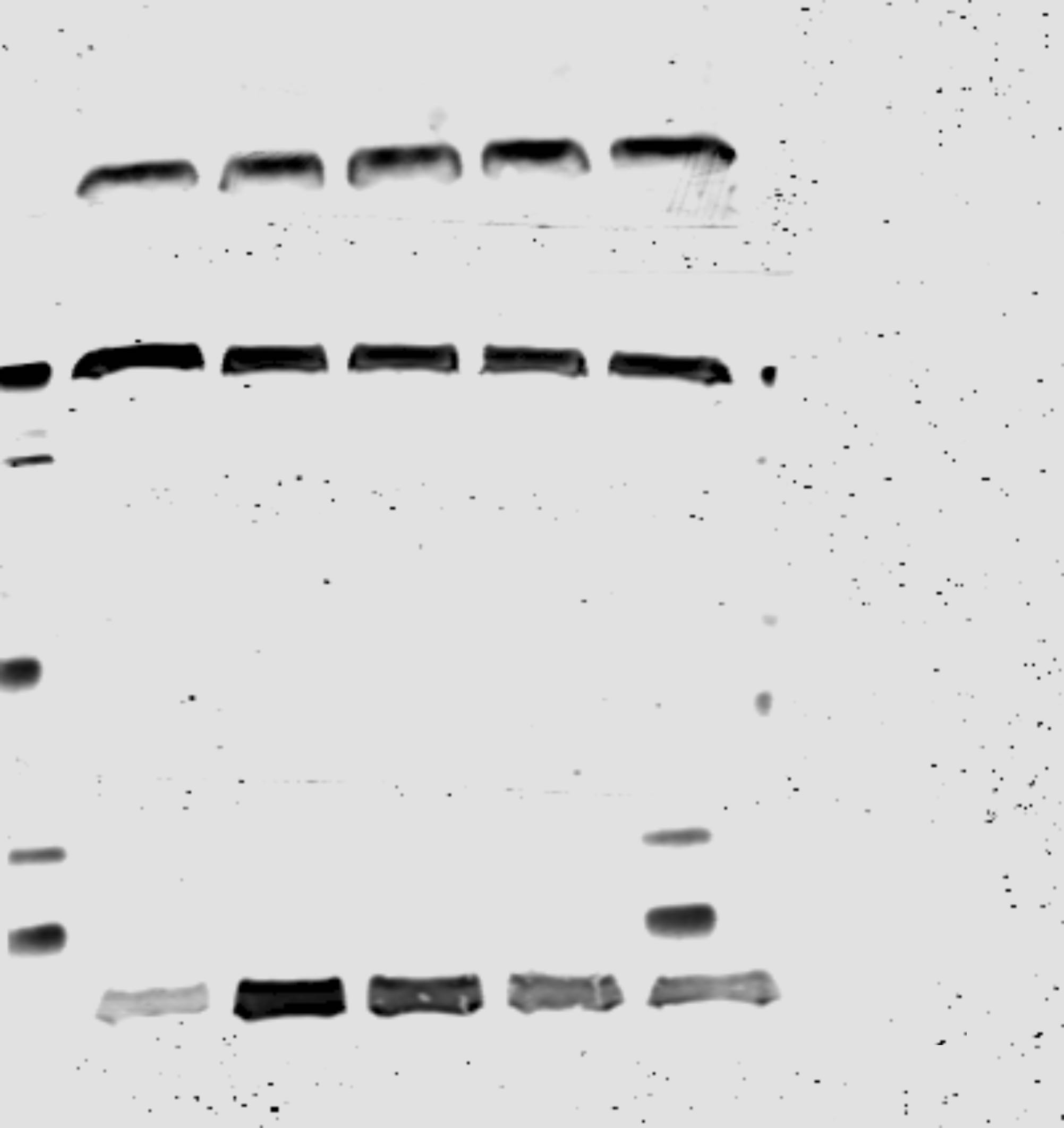


P-P65 65 kDa


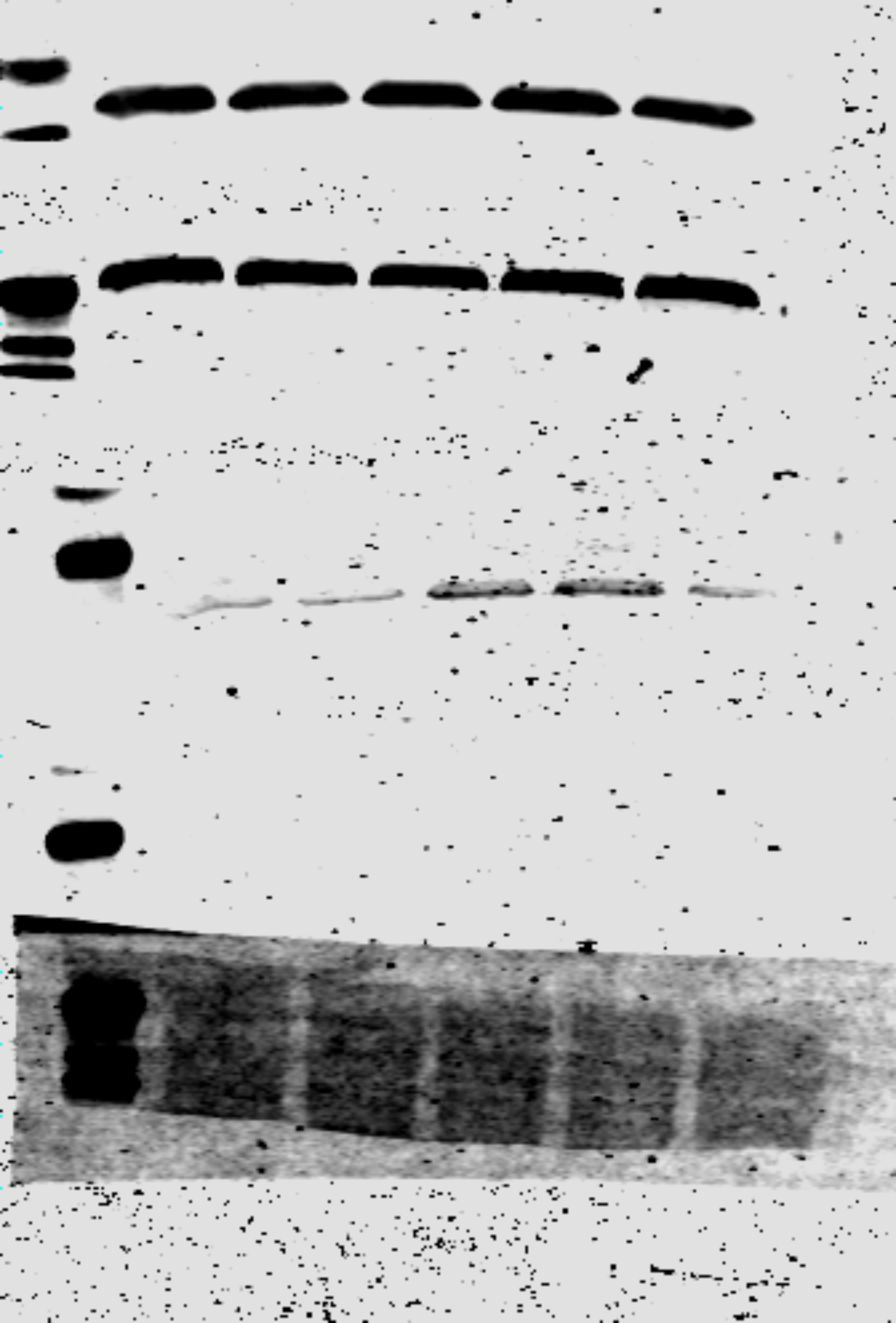
 55

40

GAPDH 37 kDa

35

con

lps

5 μg/mL

10 μg/mL

20 μg/mL

Figure Ⅱ. Original western blot


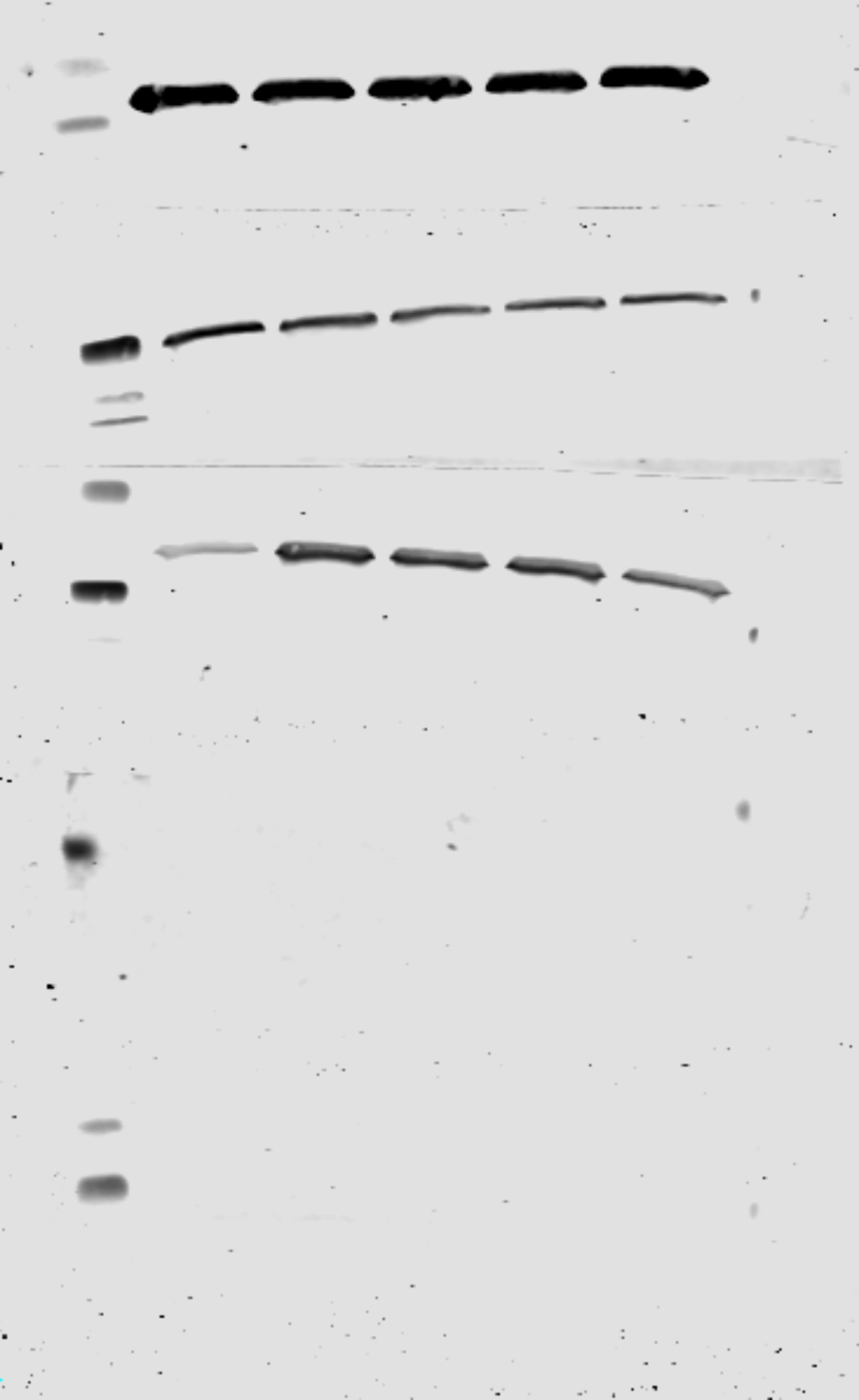


180

IKKβ 87 kDa 130

100


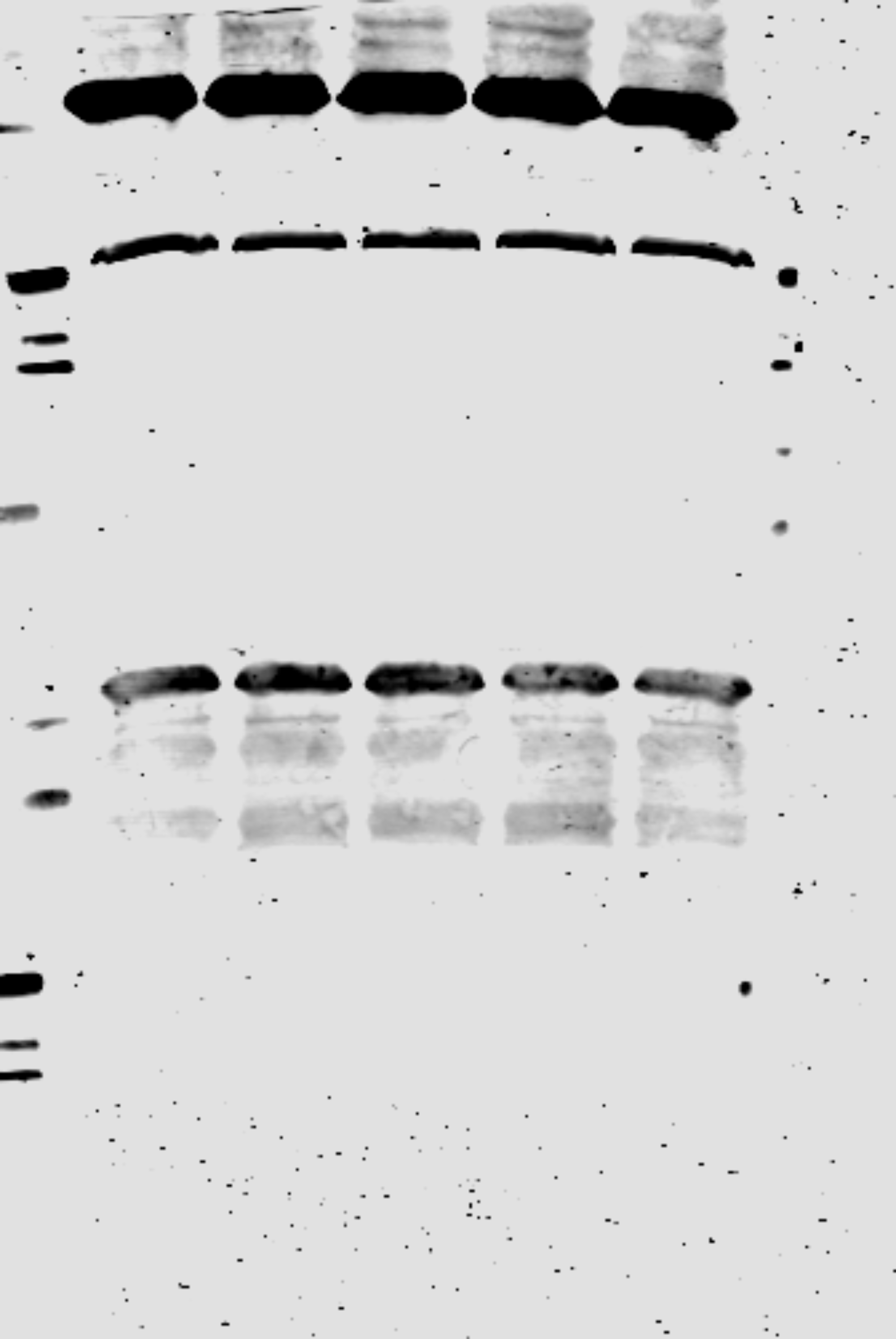


180

IKKα 85 kDa 130

100


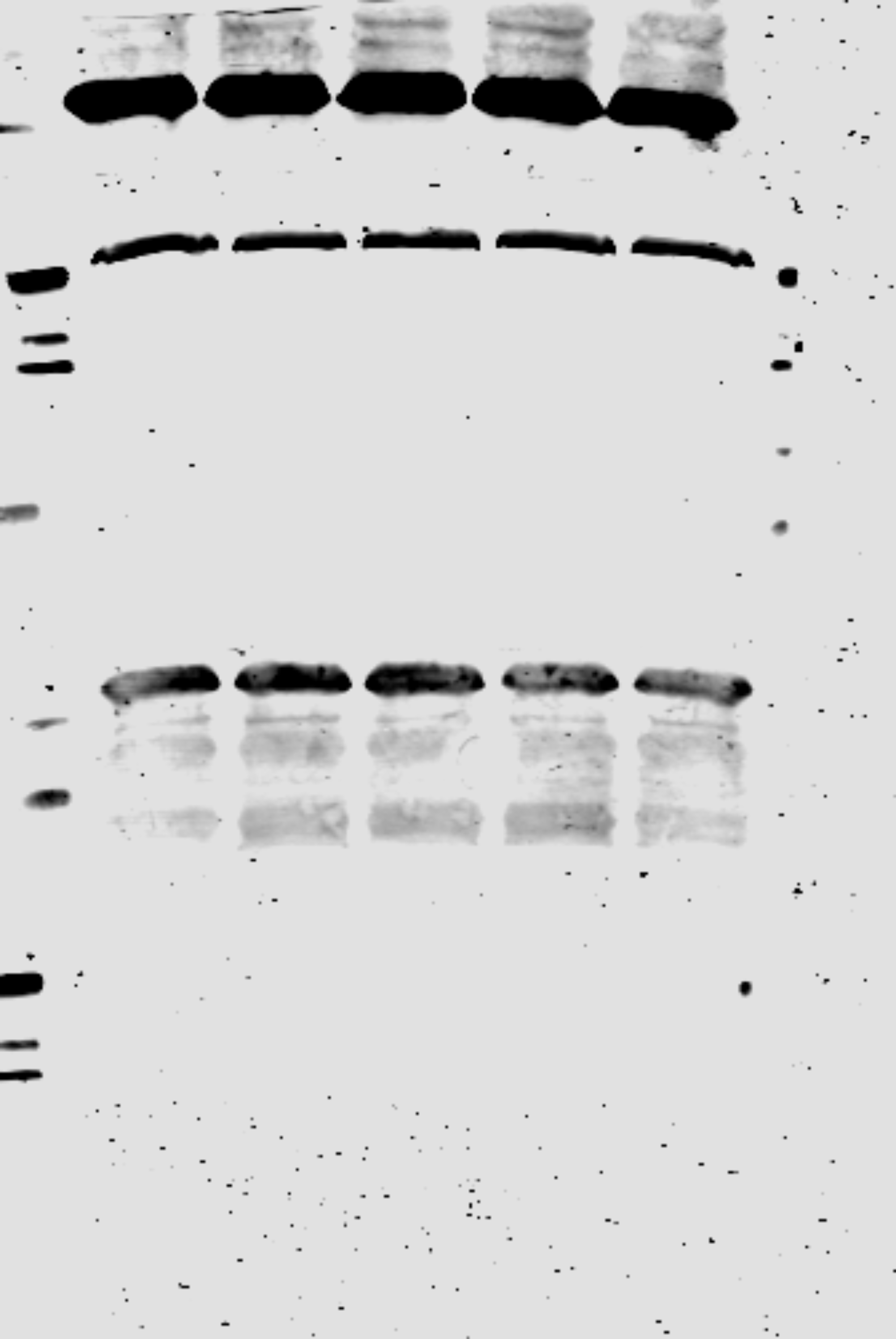


P65 65 kDa

55


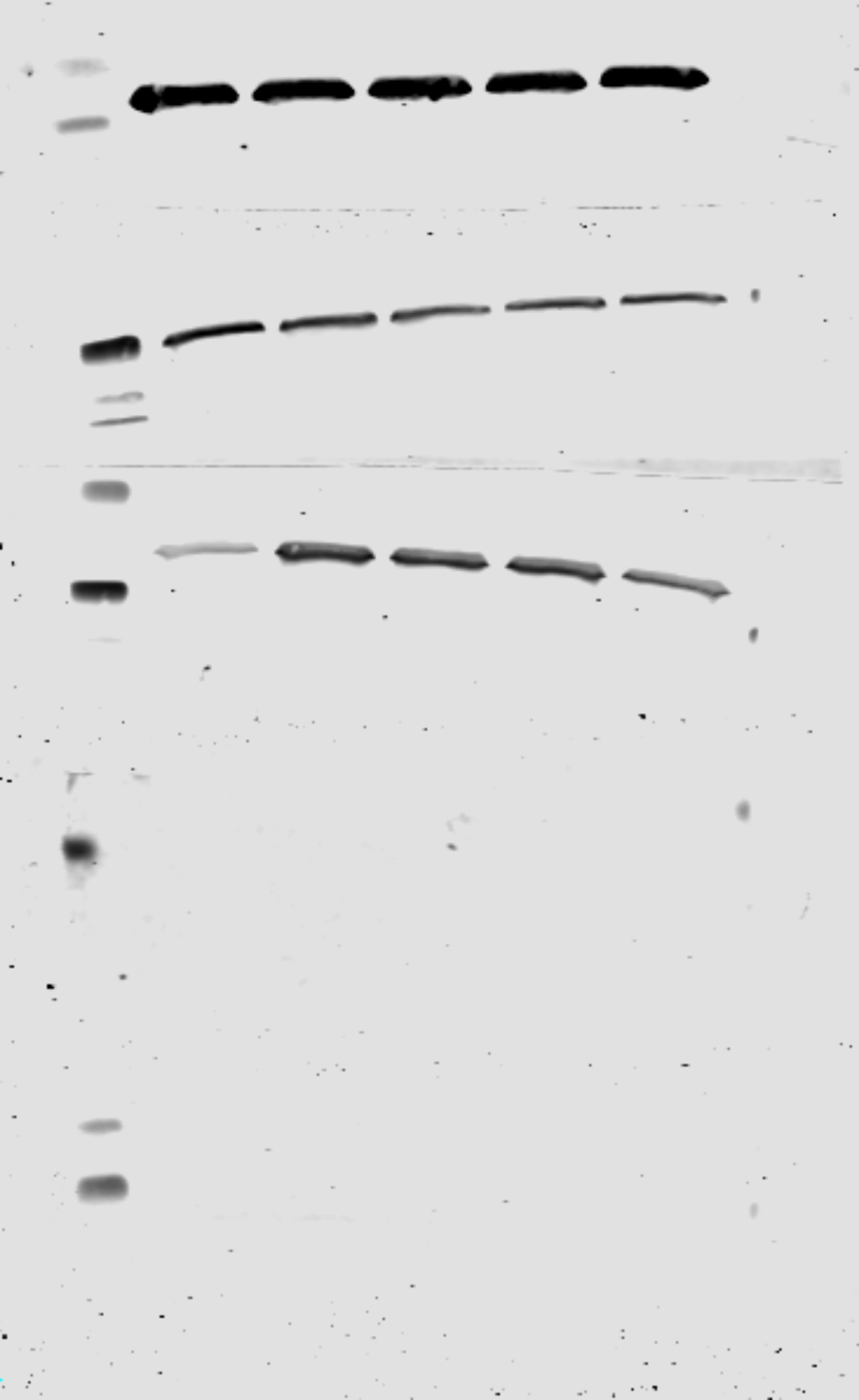
 70

P-P65 65 kDa

55


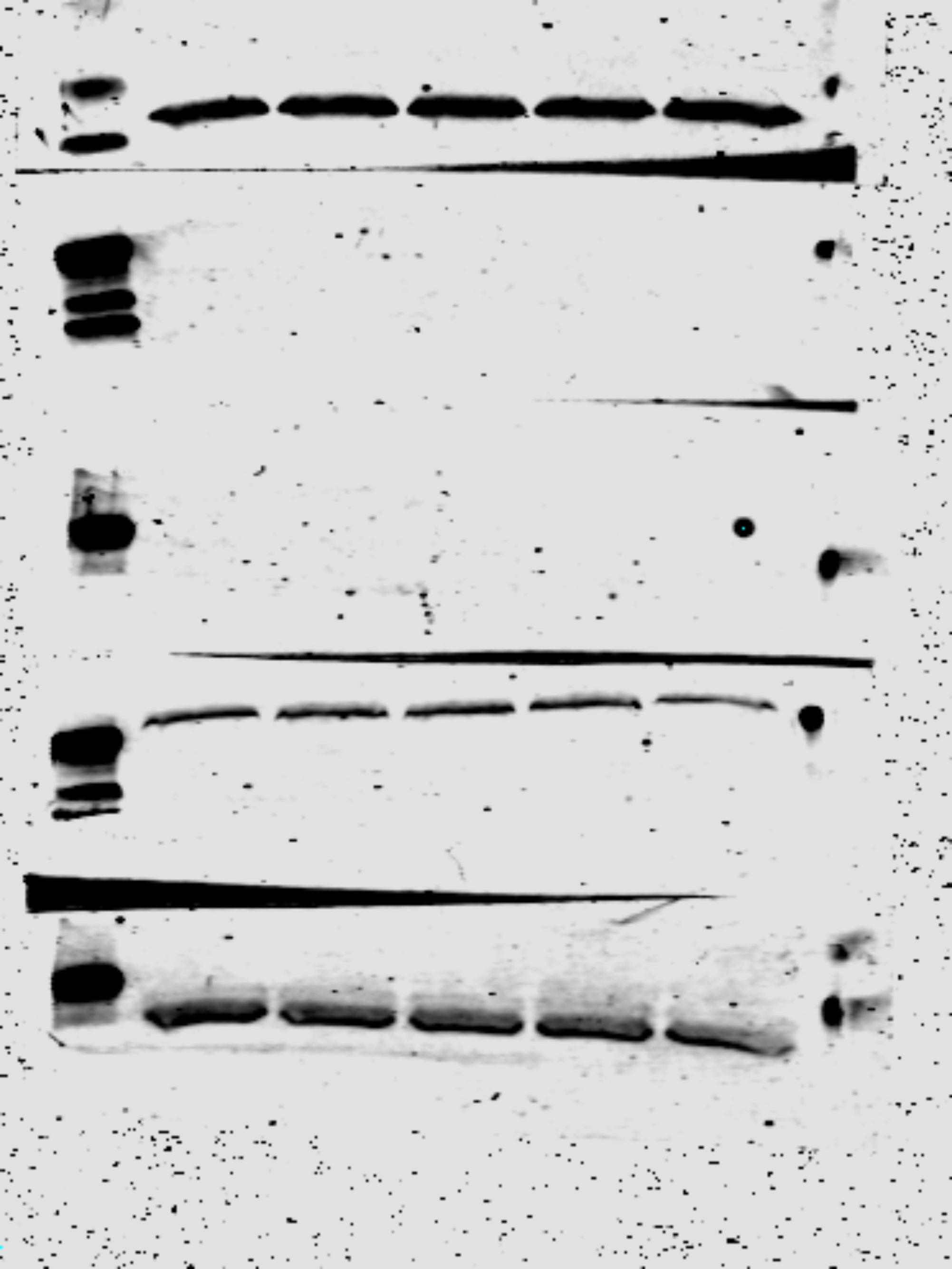


70

TRAF6 60 kDa


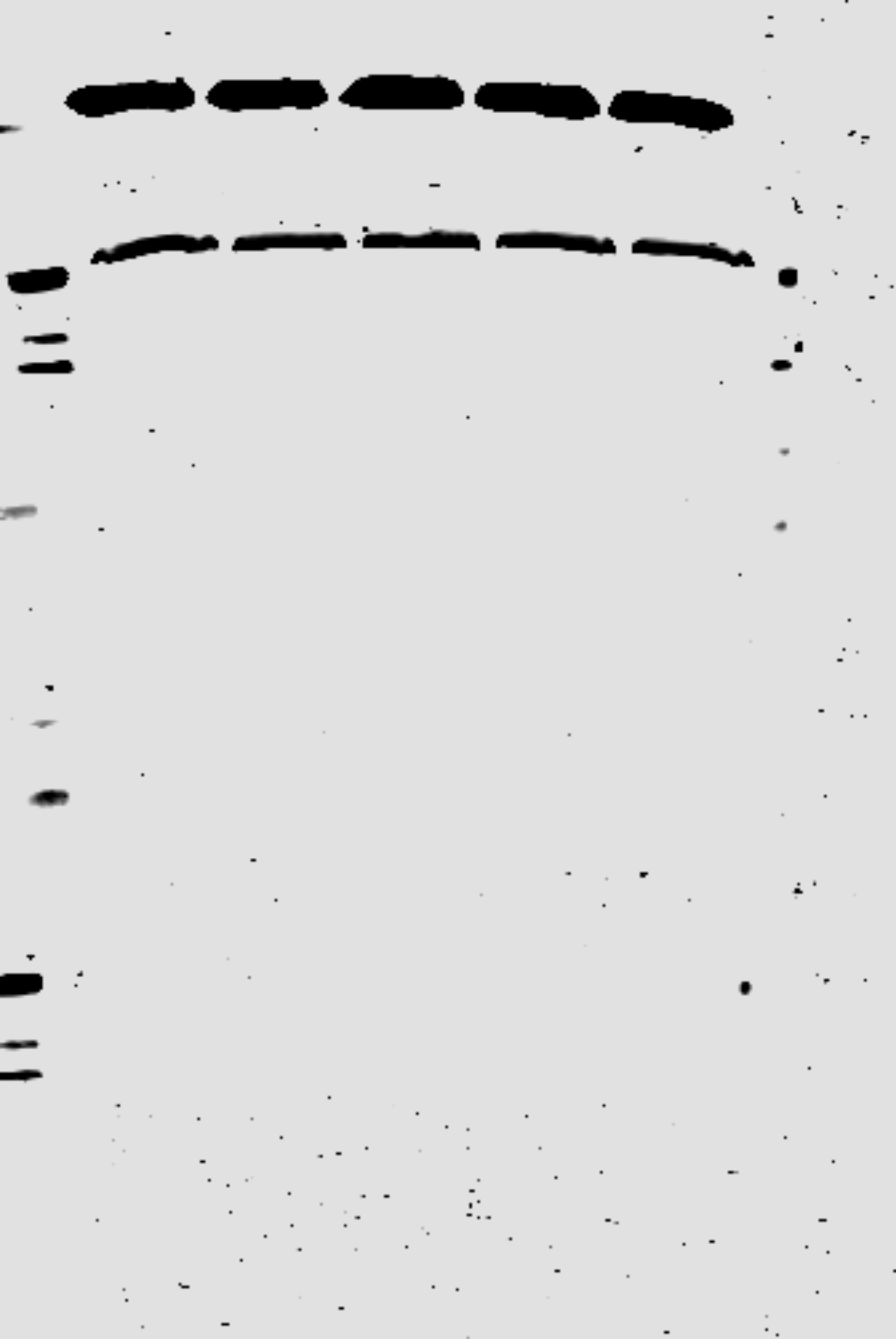


GAPDH 37 kDa 35

con

lps

5 μg/mL

10 μg/mL

20 μg/mL

Figure Ⅲ. Original western blot





180

IKKβ 87 kDa 130

100


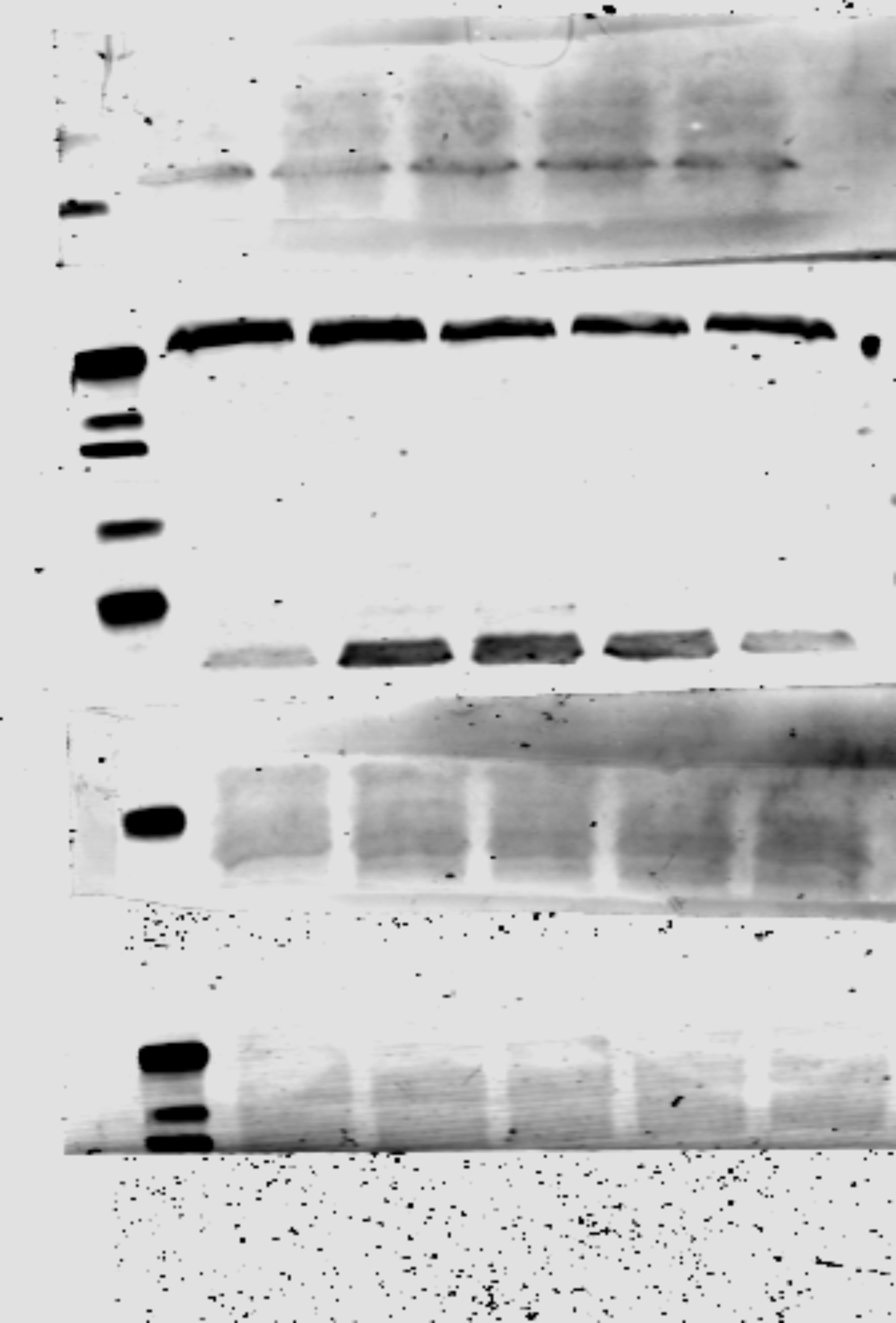


180

IKKα 85 kDa 130

100


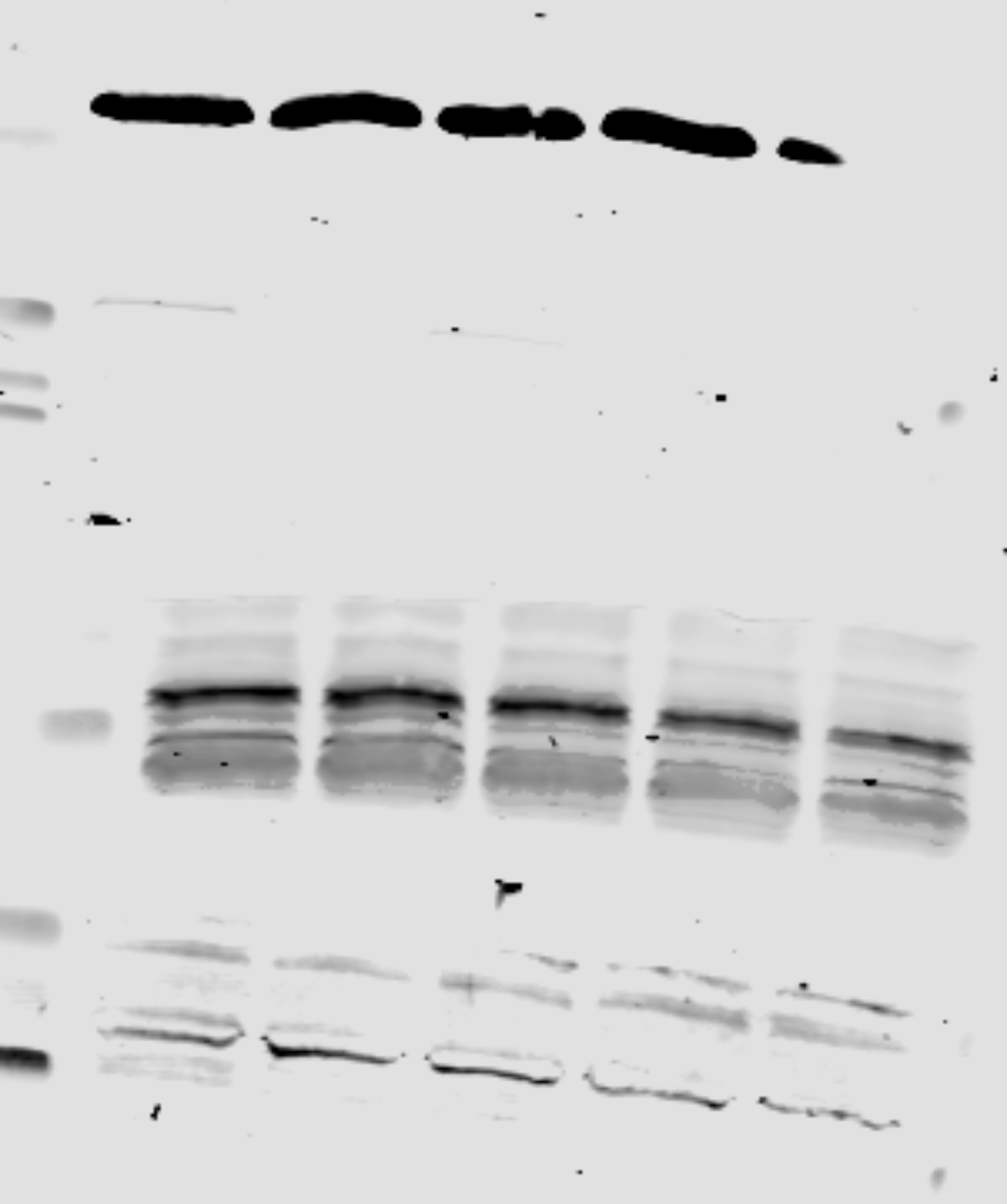
100

Zap70 70 kDa

70


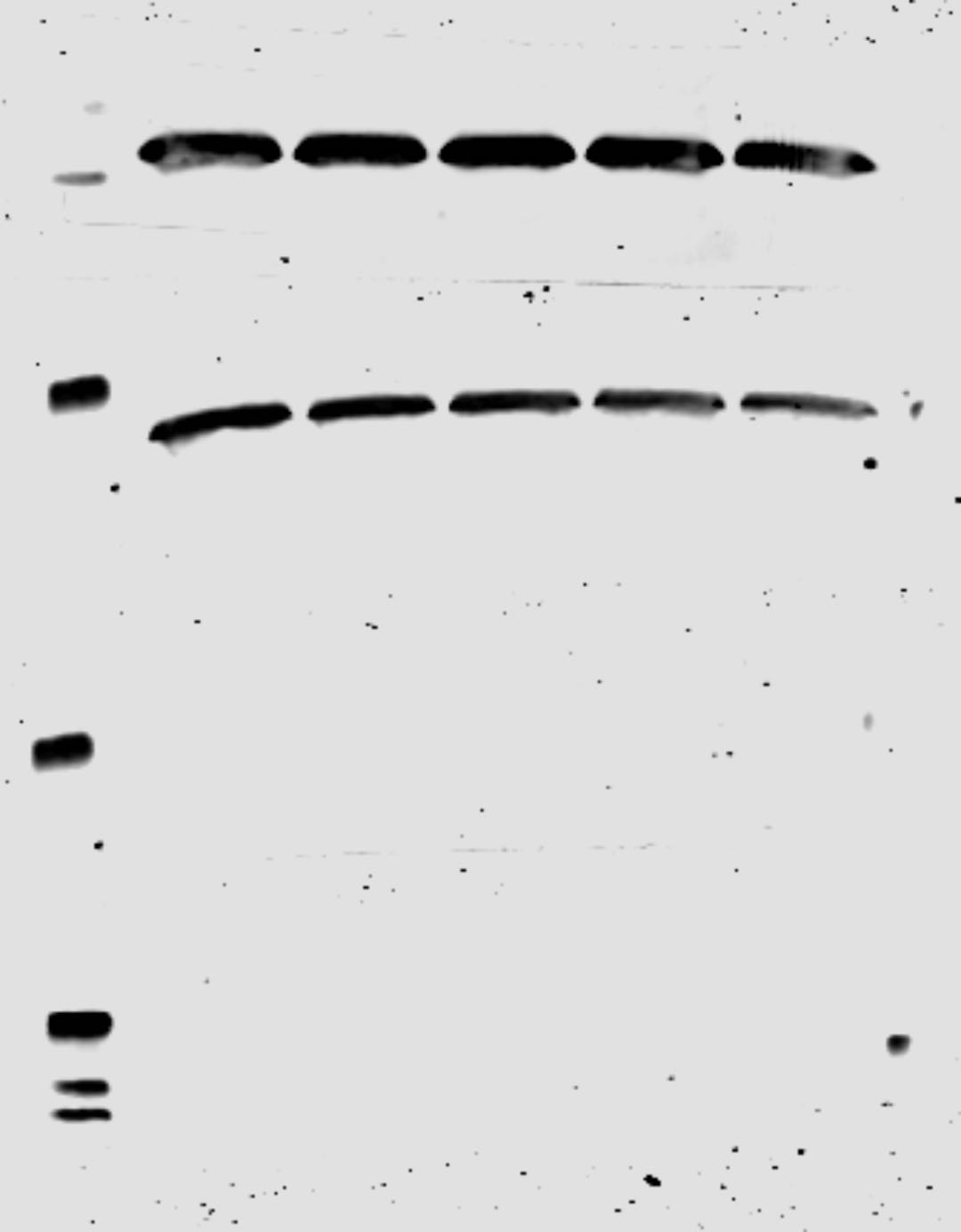


P65 65 kDa 70


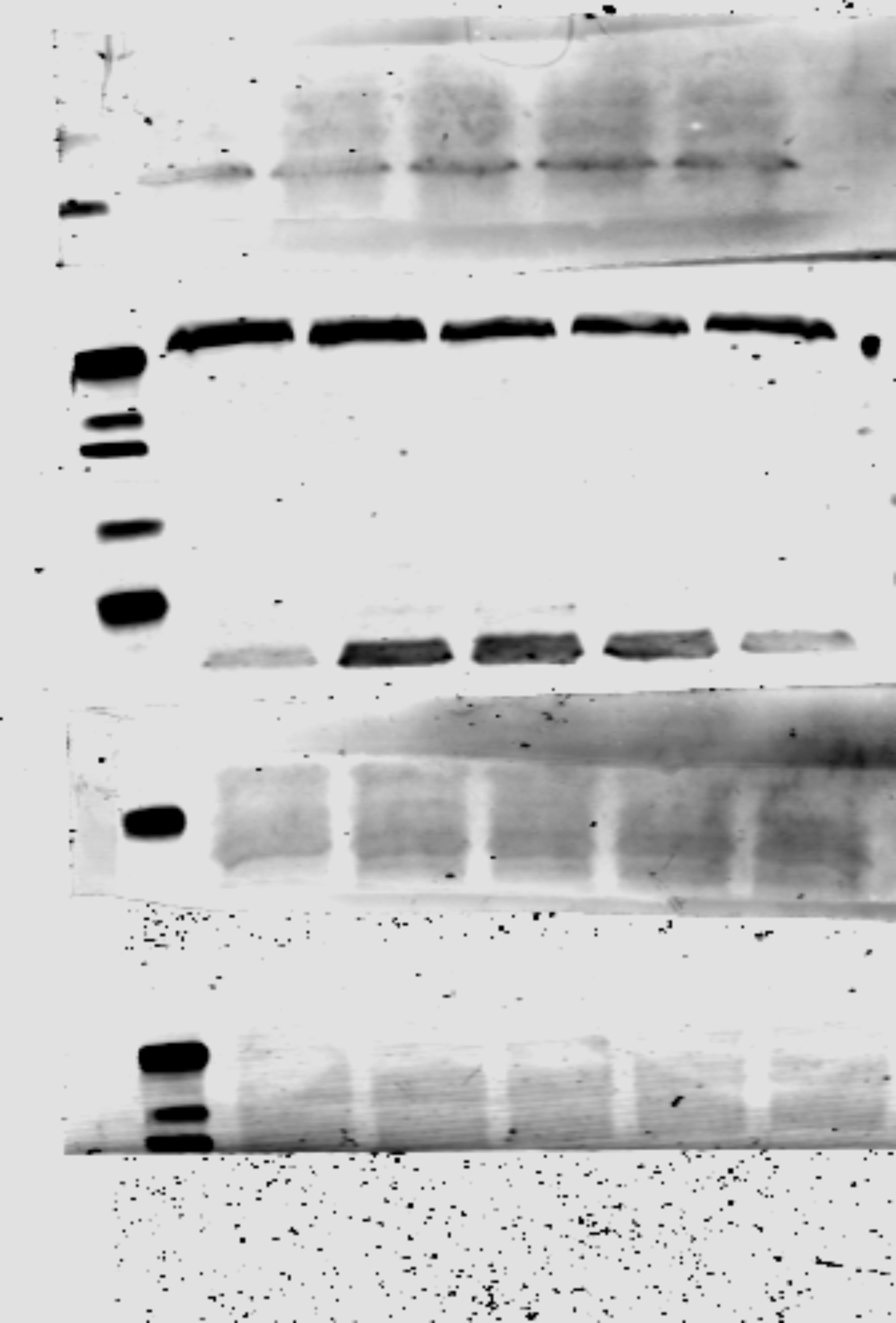
 100

P-P65 65 kDa

70


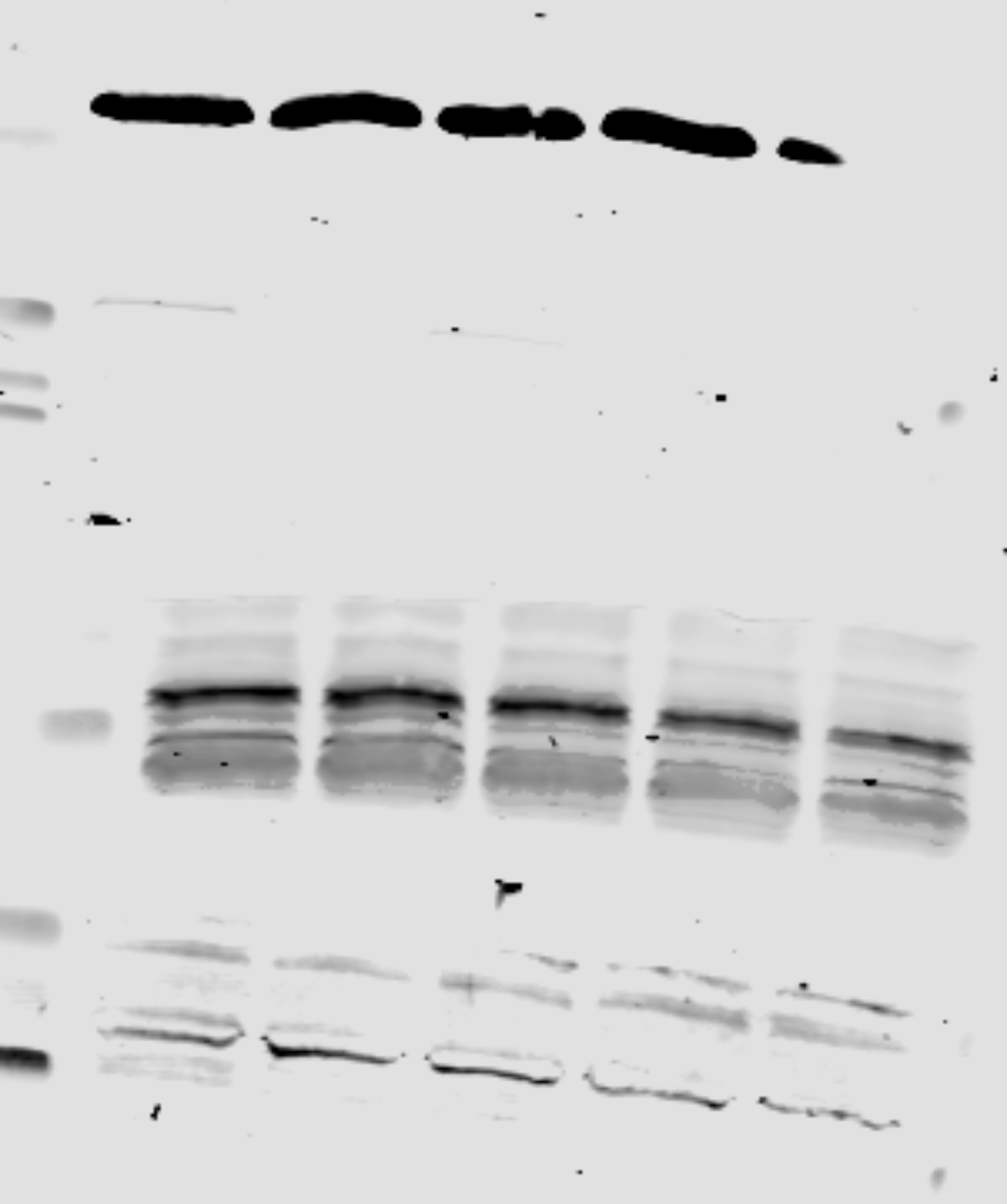
LCK 56 kDa

55


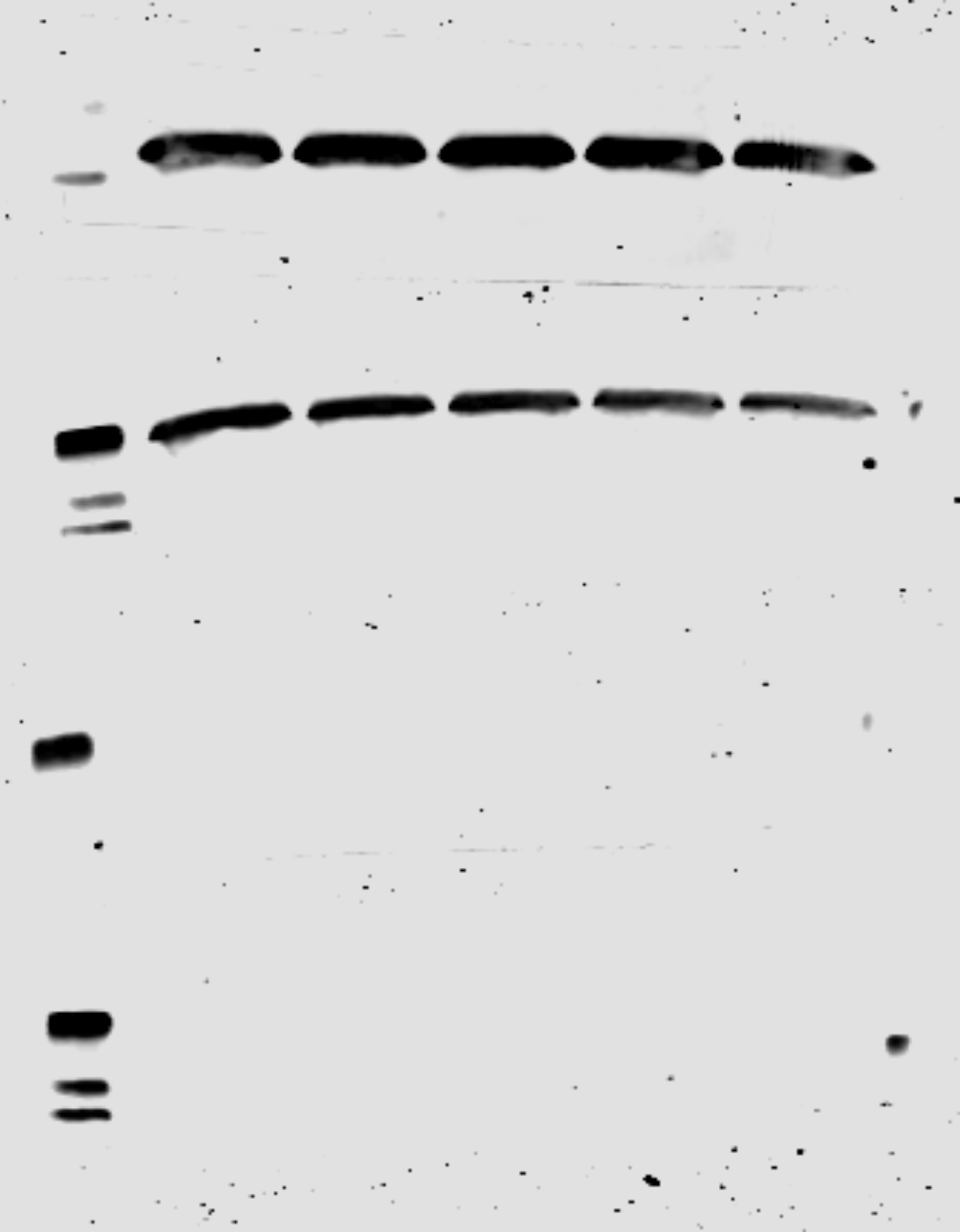


40

GAPDH 37 kDa

35

con

lps

5 μg/mL

10 μg/mL

20 μg/mL
