# Supplementary material for: Study on the anti-inflammatory effects and mechanisms of gentisic acid based on the LPS-induced RAW264.7 cell inflammation model and the oxazolone-induced zebrafish inflammation model
Source: Front Pharmacol. 2026 Jun 26;17:1837686. doi: 10.3389/fphar.2026.1837686 (PMC13350250; doi:10.3389/fphar.2026.1837686)
Supplement: Supplementary file 2 [file Image1.pdf]

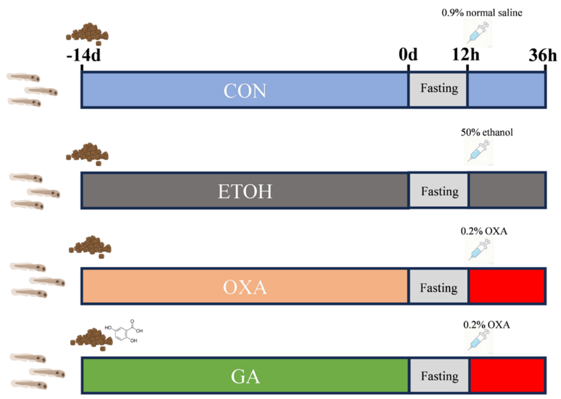

Figure S1. The experimental design for the OXA-induced zebrafish intestinal inflammation model.
